# Natural Compounds as Target Biomolecules in Cellular Adhesion and Migration: From Biomolecular Stimulation to Label-Free Discovery and Bioactivity-Based Isolation

**DOI:** 10.3390/biomedicines9121781

**Published:** 2021-11-26

**Authors:** Beatrix Péter, Imre Boldizsár, Gábor M. Kovács, Anna Erdei, Zsuzsa Bajtay, Alexandra Vörös, Jeremy J. Ramsden, Ildikó Szabó, Szilvia Bősze, Robert Horvath

**Affiliations:** 1 Nanobiosensorics Group, Research Centre for Energy Research, Institute for Technical Physics and Materials Science, Konkoly-Thege u 29-33, 1120 Budapest, Hungary; aavoros@gmail.com (A.V.); r74horvath@gmail.com (R.H.); 2 Department of Plant Anatomy, Institute of Biology, Eötvös Loránd University, 1117 Budapest, Hungary; boldizsarimi@gmail.com (I.B.); gaborm.kovacs@ttk.elte.hu (G.M.K.); 3 Department of Pharmacognosy, Semmelweis University, Üllői út 26, 1085 Budapest, Hungary; 4 Plant Protection Institute, Centre for Agricultural Research, Hungarian Academy of Sciences, 1022 Budapest, Hungary; 5 Department of Immunology, Eötvös Loránd University, 1117 Budapest, Hungary; anna8erdei@gmail.com (A.E.); bajtay.zsuzsanna@ttk.elte.hu (Z.B.); 6 MTA-ELTE Immunology Research Group, Eötvös Loránd Research Network (ELKH), Eötvös Loránd University, 1117 Budapest, Hungary; 7 Clore Laboratory, University of Buckingham, Buckingham MK18 1EG, UK; jeremy.ramsden@buckingham.ac.uk; 8 MTA-ELTE Research Group of Peptide Chemistry, Eötvös Loránd Research Network (ELKH), Institute of Chemistry, Eötvös Loránd University, 1117 Budapest, Hungary; szaboi8@gmail.com (I.S.); szilvia.bosze@gmail.com (S.B.); 9 National Public Health Center, Albert Flórián út 2-6, 1097 Budapest, Hungary

**Keywords:** natural compound, cell adhesion, movement, CAM, integrin, viability, biosensors, preparation, isolation, SARS-CoV-2

## Abstract

Plants and fungi can be used for medical applications because of their accumulation of special bioactive metabolites. These substances might be beneficial to human health, exerting also anti-inflammatory and anticancer (antiproliferative) effects. We propose that they are mediated by influencing cellular adhesion and migration via various signaling pathways and by directly inactivating key cell adhesion surface receptor sites. The evidence for this proposition is reviewed (by summarizing the natural metabolites and their effects influencing cellular adhesion and migration), along with the classical measuring techniques used to gain such evidence. We systematize existing knowledge concerning the mechanisms of how natural metabolites affect adhesion and movement, and their role in gene expression as well. We conclude by highlighting the possibilities to screen natural compounds faster and more easily by applying new label-free methods, which also enable a far greater degree of quantification than the conventional methods used hitherto. We have systematically classified recent studies regarding the effects of natural compounds on cellular adhesion and movement, characterizing the active substances according to their organismal origin (plants, animals or fungi). Finally, we also summarize the results of recent studies and experiments on SARS-CoV-2 treatments by natural extracts affecting mainly the adhesion and entry of the virus.

## 1. Introduction

Natural medicines, extracted from herbs and other living sources such as serpent venoms, have been used by humans since the earliest times. In contrast, the use of mineral substances as medicines was an innovation of Paracelsus introduced as recently as the 16th century AD. Today, there is renewed interest in natural substances for curing illnesses, both directly and as inspirations for manufactured pharmaceuticals (for example, an anti-malaria agent extracted from the sweet wormwood plant [1]). Many people prefer to take mixtures of herbs as alternatives to industrial medicine, which often causes deleterious side effects [2]. Plants (and fungi) synthesize a large number of specific compounds called secondary metabolites (SMs). The function of SMs in plants and fungi is not fully understood; however, many of these compounds can be used for medicinal purposes. They have special significance in cancer therapy: among compounds introduced since the 1940s, almost 50% were isolated, purified SMs or their semi-synthetic derivatives [3]. 

The study of the mode of action of natural substances at the cellular and molecular levels only began a few years ago with the advent of modern techniques. Compared to manufactured pharmaceuticals systematic scientific evidence for the efficacy and safety of natural substances is generally still lacking [2]. When studying SMs, the major challenge is to elucidate their effective targets, which are responsible for the medicinal effects of these natural compounds. In contrast with synthetic drugs the target is usually preselected, and the challenge is to find a molecule that binds to the target and not the others. 

We suggest that these substances influence primarily at the cellular level. The general approach has been to apply labeling techniques to investigate effects on cell adhesion, migration, motility etc. in vitro [1]. We shall review new, label-free techniques for monitoring cell adhesion and spreading directly, without label-induced perturbation.

Cell adhesion is crucial for the assembly of individual cells into tissues [4]. It is responsible for the overall architecture of the tissue [4]. Monitoring cell adhesion and spreading is important because these processes maintain the multicellular tissue structure, and individual cell migration, survival, proliferation, differentiation, gene expression, cell-cell communication and immunity, and cancer metastasis [1]. Thus, studying the adhesion and spreading of the treated cells by natural compounds helps to understand their effects on metabolism, development and physiology.

In this review we summarize the effects of natural compounds from plants, fungi and snake venom on cellular adhesion and movement, and the methods applied to reveal these effects. Finally, we mention some possible ways to prevent or reduce symptoms of COVID-19 by applying natural compounds according to the recent literature in this topic. 

## 2. Relationship between Adhesion, Movement and Inflammation

Inflammation is typified by the accumulation of leukocytes and other mesenchymal cells in response to attractant molecules at sites of injury or infection [5,6,7]. Leukocytes become activated after being exposed to chemoattractants and are capable of adhering tightly to the endothelium [7]. Cytokines and endotoxins stimulate the endothelium to become more adhesive for leukocytes [5,7,8]. The general classes of cell adhesion molecules (CAM) are integrins, selectins, the immunoglobulin superfamily of cell adhesion molecules and cadherins [5,7,9]. Integrins and selectins on circulating leukocytes mediate their adhesion to the endothelium, whereas selectins and members of the immunoglobulin superfamily on the endothelium mediate their affinity for leukocytes [7,9]. CAMs are known to play a critical role in the recruitment of cells into various tissues and in the maintenance and regulation of the integrity of the tissues [5]. 

CAM expression is tightly regulated in normal tissue environment; however, inappropriate expression of CAMs disrupts normal cell-cell and cell-matrix interactions, facilitating tumour formation.

Integrins are prime regulators of communication between cells and their microenvironment. These evolutionarily old cellular adhesion receptors play an important role in physiological and pathological processes. These large molecules are responsible for the attachment of cells to the extracellular matrix (ECM) components and cell-to-cell interactions. Integrin heterodimers are composed of noncovalently bound α and β subunits. 

**Figure 1:**
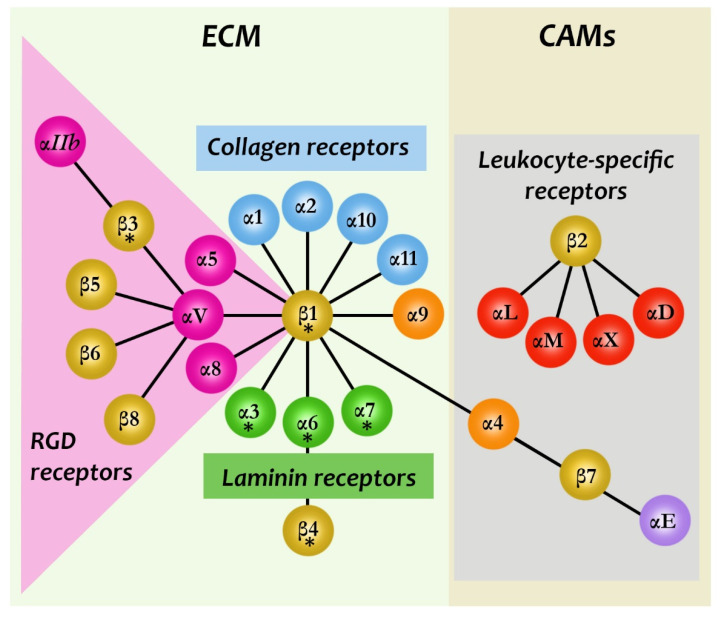
Integrin family members and their ligands. Integrins are transmembrane heterodimer molecules containing α and β subunits. The figure illustrates the association of these subunits occurring in mammalian cells. As shown, 18 α and 8 β subunits form 24 different, distinct integrins. These can be grouped in subfamilies based on evolutionary relationships (different colors of α subunits), ligand specificity and, in the case of β2 and β7 integrins, restricted expression on white blood cells. Integrins can be grouped into two larger classes that bind to cell surface cell adhesion molecules (CAMs) and ECM ligands. They can be further classified as collagen-binding integrins (α_1_β_1_, α_2_β_1_, α_10_β_1_, and α_11_β_1_), RGD-recognizing integrins (α_5_β_1_, α_V_β_1_, α_V_β_3_, α_V_β_5_, α_V_β_6_, α_V_β_8_, and α_IIb_β_3_), laminin-binding integrins (α_3_β_1_, α_6_β_1_, α_7_β_1_, and α_6_β_4_), and leukocyte integrins (α_L_β_2_, α_M_β_2_, α_X_β_2_, and α_D_β_2_). The β_2_ integrin subunit (CD18) can pair with one of the four α subunits (α_L_-CD11a, α_M_-CD11b, α_X_-CD11c, and α_D_-CD11d) [16]. The α4β1 and α9β1 integrins recognize fibronectin and VCAM-1. The β2 and β7 integrins are restrictedly expressed by leukocytes). Asterisks show the alternatively spliced cytoplasmic domains. This figure is based on the study of Hynes [10] and Yue et al. [17]. (RGD, Arg-Gly-Asp sequence; VCAM-1, a vascular cellular adhesion molecule-11).

In vertebrates the integrin family is composed of 18 α subunits and 8 β subunits that form 24 different heterodimeric complexes [10] (Figure 1). The integrins can be grouped into subgroups based on their subunit composition and ligand-binding specificity. 

A subgroup of integrins (8 out of 24) recognize proteins (such as fibronectin and vitronectin) that contain the Arg-Gly-Asp (RGD) sequence. The collagen binding integrins α1β1, α2β1, α10β1, and α11β1 are able to recognize the triple-helical GFOGER collagen sequence. The laminin receptors α3β1, α6β1 and α7β1 mediate adhesion to basement membrane laminins. Vertebrates also have leukocyte-specific integrins that mediate cell-cell adhesion. The β2 integrins are present on the cell surface in a normally inactive state, in which they do not bind ligands. An activation signal like an inflammatory stimulus results in a conformational change of the integrin, increasing its ability to bind the ligand. Activated β2 integrins play an important role in cellular adhesion, migration and phagocytosis.

The lack of the β subunit can block preimplantation development (β1) and perinatal lethality (β8) and is involved in various defects of leukocyte function (β2, β7), inflammation (β6), hemostasis and angiogenesis (β3). Integrins frequently intercommunicate; that is, they are able to activate or inhibit each other’s function [10].

The CD11/CD18 β2 integrins are specifically expressed on circulating leukocytes, and play a significant role in fast adhesion to endothelial cells [7,9]. The intercellular adhesion molecule-1 (ICAM-1) and vascular cellular adhesion molecule-1 (VCAM-1) are well-characterized endothelial cell ligands for CD11/CD18 [7,11]. The endothelial cell surface quantity of molecules like ICAM-1 is increased with the release of some proinflammatory cytokines from lymphocytes; an environment is thereby generated in which the leukocytes are more chemoreceptive to arterial walls during inflammatory processes [5]. Recruitment of arterial leukocytes is a significant step in the progression of different inflammatory diseases, such as rheumatism [12,13], liver inflammation [13,14], atherosclerosis [13,14], and inflammatory bowel disease [13]. Medical plant extracts may alter pathological mechanisms via the modulation of adhesion molecules [5]; this general observation will be analyzed and systematized below. 

All morphogenetic processes are affected by cell migration, which contributes to many illnesses, including cardiovascular disease and cancer [15]. In general, cell movement begins with extension of the membrane followed by the formation of new adhesive protrusions at the front, which link the actin cytoskeleton to the substratum, generating traction forces that move the cell forwards; adhesive protrusions at the rear are simultaneously dismantled [15]. The cycle of forming and dismantling of adhesive structures drives migration [15]. Rho GTPases have an important role in this process; they regulate actin polymerization and myosin II activity and, therefore, adhesion dynamics [15].

The adhesion molecules driving migration are the same as those involved in the inflammatory response, but their expression and ligand-binding capacity depend on the stimuli.

## 3. Mechanisms of Action of Natural Compounds

### 3.1. Prestimulation with Cytokines 

**Figure 2:**
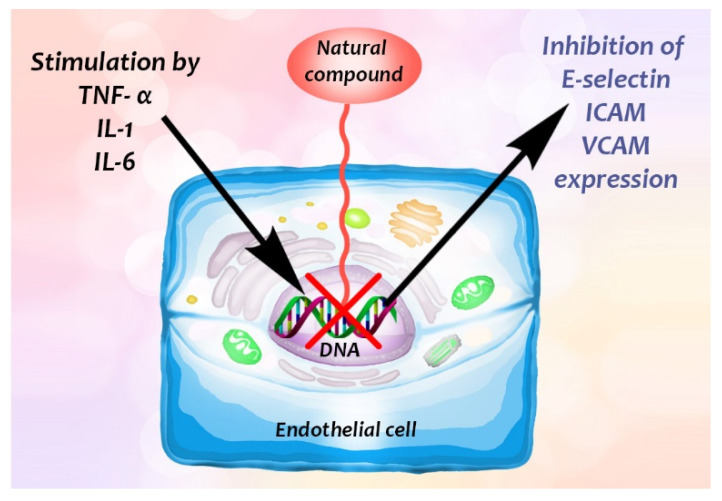
Adhesion molecule stimulation and mechanisms in endothelial cells. Inhibitory effect of a natural compound on cellular adhesion, typically of the human umbilical vein endothelial cell line (HUVEC), which are usually first treated with certain cytokines to stimulate the expression of CAMs.

**Figure 3:**
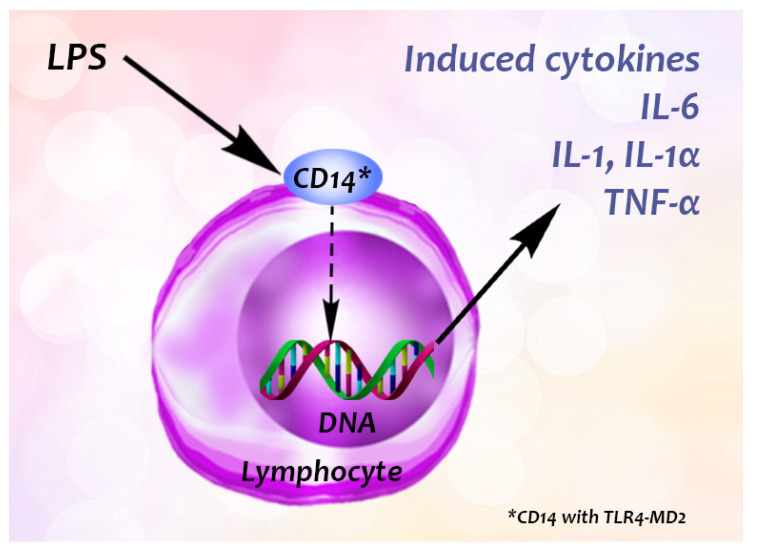
Stimulation mechanism for cytokine modulation in lymphocytes. In vivo, lipopolysaccharide (LPS, from Gram-negative bacteria) stimulates the immune response by interacting with its leukocyte membrane receptor, CD14 (with TLR4-MD2), to induce the generation of cytokines such as TNF- α, IL-1, IL-1 α, IL-6.

To demonstrate the inhibitory effect of a natural compound on cellular adhesion, cells, typically from the human umbilical vein endothelial cell line (HUVEC), are usually first treated with certain cytokines to stimulate the expression of CAM (Figure 2). In vivo, lipopolysaccharide (LPS, from Gram-negative bacteria) stimulates the immune response by interacting with its leukocyte membrane receptor, the Pattern Recognition Receptor (PRR) CD14 (with TLR4-MD2), to induce the generation of cytokines such as tumour necrosis factor α (TNF-α), interleukin-1 and -6 (IL-1, IL-6) (Figure 3). TNF-α is also involved in systemic inflammation [2,18]. It is primarily produced by activated monocytes or macrophages [19]. Note, cytokine generation, increase of expression of cytokines can also be stimulated by certain plant extracts (for example, among others, garlic (*Allium sativum*) decreases the level of IL-1α, IL-2, IL-6, IL-12, TNF- α and IFN-γ, however, increases IL-10), as summarized by Spelman, et al. [20]. After stimulation of the endothelial cells, the plant (or venom) extract is then added to them. The natural compound may downregulate expression of the adhesion molecules, resulting in the diminution of cell adhesion, and the compound has an antiinflammatory effect in consequence. The ICAMs and VCAMs are the most researched adhesion molecules [5]. We elaborate on this in the next section.

### 3.2. Inhibition of CAMs by Suppression of Their Expression (Downregulation)

The phytochemicals of olive oil (from *Olea europaea*) and red wine (from *Vitis vinifera*), oleuropein (monoterpene, seco-iridoid glucoside), hydroxytyrosol (phenylethanoid), tyrosol (phenylethanoid), elenolic acid (monoterpene, seco-iridoid) and resveratrol (stilbene) at nutritionally relevant concentrations have been shown to inhibit endothelial adhesion molecule expression (Figure 2). This provides a strongly suggestive basis for the atheroprotective property of the so-called “Mediterranean diet” [21].

Walnut *(Juglans regia*) extract and its principal active component ellagic acid decreased the stimulated endothelial expression of ICAM-1 and VCAM-1, indicating a mechanism for the known antiatherogenic and osteoblastic activity of the substance [18]. A walnut-enriched diet may therefore indeed be cardioprotective and inhibit osteoporosis [18]. 

Curcumin (diphenylheptanoid) from the *Curcuma longa* rhizome also downregulated the expression of adhesion molecules and, hence, monocyte adhesion [13]. Saponin (triterpene) astagaloside IV from Mongolian milkvetch *(Astagalus membranaceus*) decreased the LPS-induced expression of VCAM-1 and E-selectin on the surface of HUVEC, hence this Chinese traditional medicinal herb is predicted to have anti-inflammatory efficacy; however, ICAM-1 was not affected [22]. 

*Tripertygium wilfordii* is a vine-like plant that grows in south China, and in the Chinese pharmacopoeia the extract from its root is prescribed for treating long-term rheumatoid arthritis and systemic lupus erytnematosus [7,8]. Chang et al., applied IL-1α to stimulate HUVEC cells; treatment with a high concentration (50 ng/mL) of the herb extract (containing wilforonide, alkaloids, diterpenes, triterpenes, b-sitosterol, daucosterol, dulcitol and glycosides [7,23]) had a significant inhibitory effect on both the expression and secretion of the cellular adhesion molecules, and thus may be a potential therapeutic agent for the treatment of inflammatory diseases [7].

### 3.3. Mechanism of Downregulation

The downregulation of CAMs by natural products is achieved by inhibiting their gene expression. 

**Figure 4:**
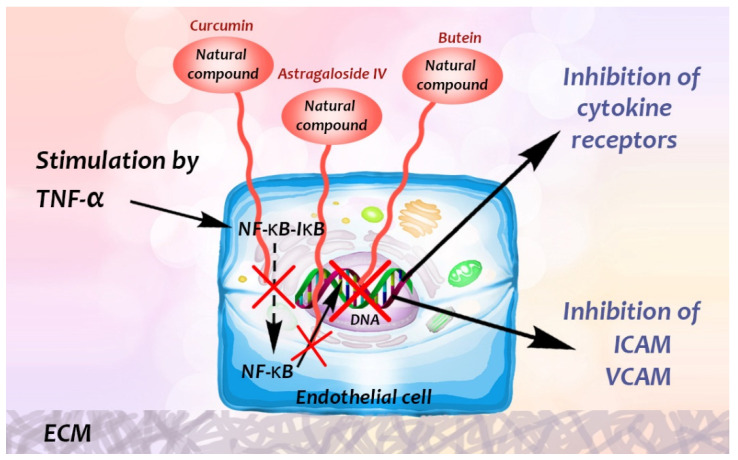
The NF-κB pathway in endothelial cells. The compounds shown to have anti-inflammatory effects in vivo completely annulled LPS- and TNF-α-triggered nuclear translocation of NF-κB and NF-κB DNA-binding activity in endothelial cells, thus the production of cytokine receptors and ICAM, VCAM is inhibited.

The molecular details involve the uptake of the natural product by the cytoplasm followed by interaction between the compound and transcription factors for adhesion molecule genes. Activation of the transcription factor nuclear factor κB (NF-κB), is mediated by the proinflammatory cytokines mentioned above, for instance TNF-α, and triggers gene expression of adhesion molecules (Figure 4). NF-κB binding sites are found in the promoter region of E-selectin, ICAM-1 and VCAM-1 [13,24,25,26,27]. NF-κB is in the cytoplasm in inactive form, complexed to IκB “nuclear factor of κ light polypeptide gene enhancer in B-cells inhibitor” [13]. When cells are stimulated with TNF-α, the IκB is phosphorylated, ubiquitinated, and degraded. The thereby activated NF-κB translocates to the nucleus and transcriptionally up-regulates cytokine receptors as well as the adhesion molecules [13,26,28,29].

The expression and function of integrins on various immune cells are summarized in Table 1.

Kawasaki and co-workers demonstrated that hot-water extract of *Curcuma longa* also suppressed the phosphorylation and degradation of IκBα in endothelial cells [13]. Another extract, the triterpene saponin astragaloside IV (3-O-β-D-xylopyranosyl-6-O-β-D-glucopyranosylcycloastragenol) from the Chinese herb *Astragalus membranaceus,* shown to have anti-inflammatory effects in vivo, completely annulled LPS- and TNF-α-triggered nuclear translocation of NF-κB and NF-κB DNA-binding activity in endothelial cells [22] (Figure 4), furthermore, it has shown that it has antioxidative stress, antiapoptosis, and antifibrosis activities, both in vitro and in vivo [34,35].

Recent studies have shown that astragaloside IV (AS-IV) administration ameliorates diabetic neuropathy in streptozotocin (STZ)-induced diabetic rats via an anti-inflammatory mechanism [36], inhibits endoplasmic reticulum stress [37], and protects podocytes [34,38]. However, the effect and mechanism of AS-IV on diabetic neuropathy induced by type 2 diabetes remain unknown [34]. Flavonoid quercetin attenuates TNF-α-induced ICAM-1 and MMP-9 expression in ARPE-19 cells via the MEK1/2–ERK1/2 and PKCδ–JNK1/2–c-Jun or NF-κB pathways [39]. Flavonoid apigenin significantly suppressed the TNF-α-stimulated upregulation of VCAM-1-, ICAM-1-, and E-selectin-mRNA to the basal levels [40]. Simple phenolic compound salicin inhibits IL-1β-induced production of pro-inflammatory cytokines such as TNF-α, IL-6, and monocyte chemoattractant protein-1 (MCP-1), vascular adhesion molecules such as (ICAM-1 and VCAM-1, and high-mobility group protein 1 (HMGB-1)) [41].

Lignan-type active compounds manassantin A and B, dineolignan compounds, inhibited the PMA-induced ICAM-1/LFA-1-mediated homotypic aggregation of the HL-60 cells without cytotoxicity, with MIC values of 1.0 and 5.5 nM, respectively. Even though these compounds did not affect the adhesion of ICAM-1 to LFA-1, they inhibited PMA-induced ICAM-1 expression in HL-60 cells in a dose-dependent fashion. These results suggest that manassantin A and B inhibit cell aggregation through downregulation of ICAM-1 expression [42].

HUVECs treated with sesquiterpene α-iso-cubebene showed markedly suppressed TNF-α-induced mRNA expression of VCAM-1 and E-selectin, but little alteration in ICAM-1 mRNA expression. α-iso-cubebene treatment also significantly decreased the TNF-α-induced cell surface and total protein expression of VCAM-1 and E-selectin without affecting ICAM-1 expression [43].

Diterpene andrographolide significantly reduced E-selectin expression of activated endothelial cells, and inhibited E-selectin expression at the mRNA level [44].

In vitro, triterpene saponine dioscin decreased monocyte adhesion to TNF-α-treated HUVECs by reducingvascular cell adhesion molecule-1 (VCAM-1) and intercellular adhesion molecule 1 (ICAM-1) expression and inhibiting endothelial lipase (EL) expression in TNF-α-treated HUVECs and macrophages by blocking the NF-kB pathway [45].

Ethyl 3′,4′,5′-trimethoxycinnamate and piperine are the two active principles of *Piper longum*. Using primary human umbilical vein endothelial cells, Kumar et al. [46] evaluated the activities of ethyl 3′,4′,5′-trimethoxycinnamate on TNF-α-induced expression of cell adhesion molecules, ICAM-1, VCAM-1 and E-selectin, which play key roles in controlling various inflammatory diseases. Both compounds inhibited the TNF-α-induced expression of ICAM-1 [46].

*Ganoderma lucidum*, a medicinal mushroom, has been used in traditional Chinese medicine to prevent and treat various diseases, for example cancer [47]. A polysaccharide derived from the fungus interacted with cell surface proteins and β1-integrin expression was diminished, while β-actin expression was not affected [47].

We summarize the different modes of pathway intervention involving gene expression in Table 2.

### 3.4. Intervention at the ECM

**Figure 5:**
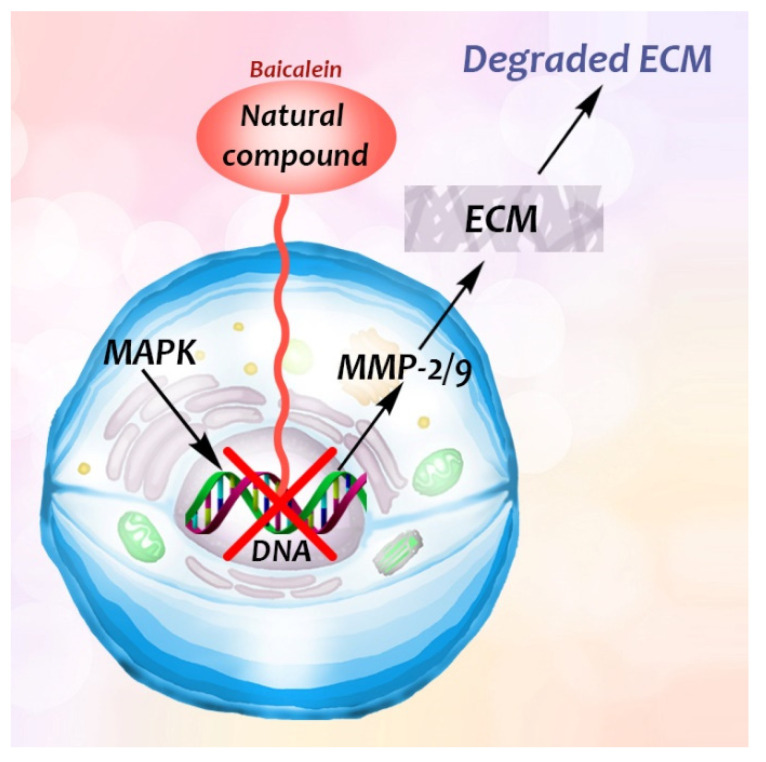
The interaction between the ECM, MMPs and natural compounds in breast carcinoma. Baicalein blocked the expression of MMP-2/9, thus the degradation of the extracellular matrix is also inhibited.

Flavonoid baicalein, derived from the root of *Scutelaria baicalensis*, a widely used Chinese herbal medicine that has been used in anti-cancer and anti-inflammatory therapy [48], has an inhibitory effect on the expression of matrix metalloproteinases (MMPs) [48], which are involved in the degradation of the extracellular matrix. Destruction of basement membranes and stromal extracellular matrix is critical for favoring metastasis and invasion of malignant cells [48]. The MMPs have therefore a role in promoting tumour growth, invasion and metastasis. Treatment of human breast carcinoma (MDA-MB-231) cells with baicalein inhibited the expression of MMP-2/9, which is a result of the mitogen-activated protein kinase (MAPK) signaling pathway [48] (Figure 5).

Flavonoid (chalcone) butein (3,3,2′,4′-tetrahydroxychalcone) is an active substance found in several plants, such as Semecarpus anacardium, Dalbergia odorifera, Caragana jubata and Rhus verniciflua [49]. It has been demonstrated that it decreased leukocyte adhesion to A549 cells through the inhibition of TNF-α-induced ICAM-1 and VCAM-1 expression by inhibiting the NF-κB/MAPK/Akt signaling pathway. Butein also inhibits ROS generation [49], and may prevent TNF-α-induced airway inflammation [49] (Figure 4).

### 3.5. Inhibition of CAM Binding by Blocking Specific Cell–Surface Receptor Sites

We can distinguish three types of mechanisms inhibiting cell adhesion by blocking (B) specific receptor sites (Table 3).

In contrast to intervention at the level of protein expression, natural compounds can also specifically block cell recognition motifs, such as the amino acid triplet RGD. For example, the polyphenol EGCG from green tea has been shown to block RGD motifs (B) (Arg-Gly-Asp) and, hence, inhibit adhesion [50].

We recall that integrins are transmembrane heterodimers, a family of plasma membrane receptors that mediate adhesion of leukocytes to ECM [51]. Integrins are also involved in pathophysiological processes as well, such as genetic and autoimmune diseases, metastasis and thrombosis and, thus, integrins are important therapeutic target structures [51,52]. Blocking or disruption of binding to integrin receptors is therefore an important topic in industrial drug discovery [51,53]. Serpent venom disintegrins, a family of low molecular weight proteins, typically contain the RGD motif, and are known to block integrin activities by binding with high affinity to the integrins [51,54]. For example, rhodostomin, a snake venom from *Calloselasma rhodostoma,* blocked the integrin ανβ3 and also affected pp125 FAK phosphorylation and the actin cytoskeleton [55]. Rhodostomin contains an RGD motif that specifically inhibits the integrin-binding function [56]. It can be produced in *Pichia pastoris* (methylotrophic yeast) as well and it inhibits platelet aggregation with a *K*(I) of 78 nM as potent as native rhodostomin [56]. However, its D51E mutant blocks platelet aggregation with a *K*(I) of 49 mM [56]. Structural analysis of rhodostomin and its D51E mutant showed that they have the same tertiary fold with three two-stranded antiparallel beta-sheets [56]. Two minor differences between them were inferred from their backbone dynamics and 3D structures [56]. The docking of rhodostomin into integrin αIIbβ3 showed that between the backbone amide and carbonyl groups of the D51 residue were formed hydrogen bonds with the integrin residues R216 and R214, respectively [56]. In contrast, these hydrogen bonds were absent in the D51E mutant-integrin complex [56]. 

Another serpent venom, echistatin from *Echis carinatus*, inhibited integrin-mediated cell adhesion via selective recognition by αIIbβ3, α5β1, ανβ3 integrins [51], preventing their adhesion to the ECM. 

Not only snake venom affects integrin-mediated adhesion, but herbs as well. Epigallocatechin-gallate (EGCG) (flavonoid ester) is the main polyphenol of green tea *(Camellia sinensis)*. Many studies have shown its beneficial effect on human health [1]. The majority of them demonstrated direct effects on cell adhesion and movement. In a previous study we showed that EGCG indirectly affects HeLa cell adhesion: the cells cannot adhere onto EGCG-pre-treated model ECM coatings [50]. The polyphenol formed multilayers in poly-L-lysine polyethylene-glycol-RGD (PLL-g-PEG-RGD) chains, and blocked the RGD motifs [50]. EGCG alters the properties of mucin as well; EGCG-mucin mixtures showed that discrete particles are formed and their size increases with the ratio of EGCG to mucin [57]. Another natural compound, cistifolin (benzofuran derivative) from the rhizome of the gravel root (*Eupatorium purpureum)*, known as an anti-rheumatic herbal drug, was identified as a potent inhibitor of β1 and β2 integrin-mediated cell adhesion and, thus, has therapeutic potential for diseases where integrin adhesion molecules play a significant role [58]. 

## 4. Measurement Techniques for Monitoring Cellular Functions, Adhesion and Viability

### 4.1. Classical Techniques for Measuring Cell Viability

Experimental natural compounds are added to stimulated cell cultures to induce a CAM response, which is commonly measured by labeling methods, mainly the enzyme-linked immunosorbent assay (ELISA), Western blot, and flow cytometry. ELISA uses antibodies linked to enzymes that create a color change (e.g., by altering a dye) to identify the examined substances [13]. The Western blot is a widely used technique in biology to detect specific proteins in a sample by letting animal-derived or synthetic antibodies bind to them [13]. ELISA and Western blot techniques cannot be used for measuring cell viability directly; however, for example, Western blot can be applied as a complementary method to study the mechanism of cell death (apoptosis, autophagy markers, etc.), thus it provides a lot of information about the mechanism. Flow cytometry is usually a laser-based technique for cell sorting and counting. A wide range of fluorophores can be used in flow cytometry measurements. Fluorophores are fluorescent labels that can attach to the antibody that recognizes the target molecule of the cell [2,59]. However, impedance-based flow cytometers also exist, known as Coulter counters, which are well established label-free methods for sizing and counting cells and particles [60].

**Figure 6:**
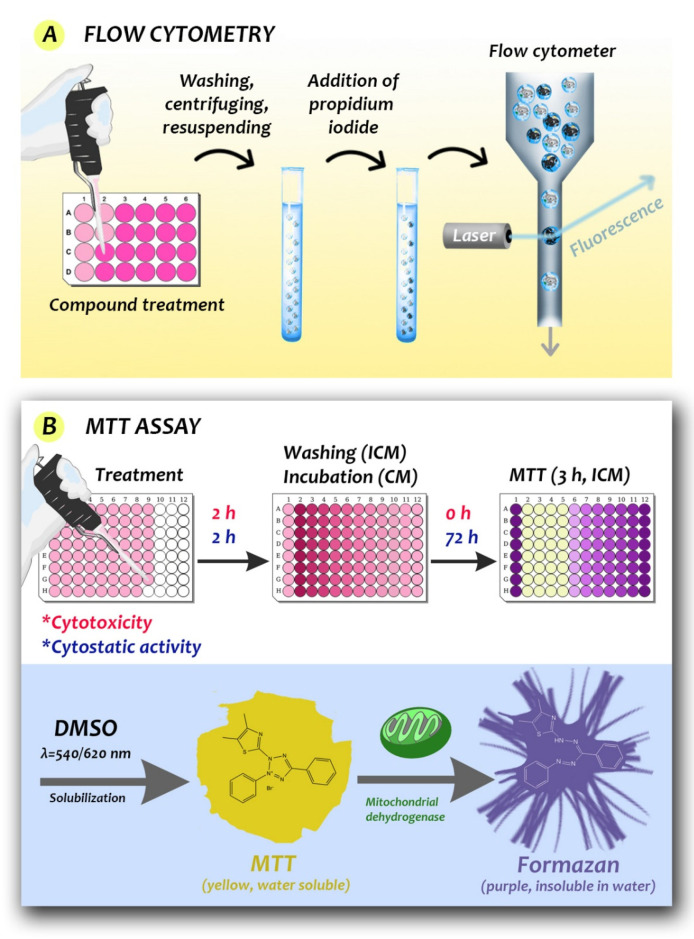
Schematic illustration of the measurement steps of flow cytometry (**A**) and tetrazolium-based colorimetric MTT viability tests (**B**). (**A**) Propidium iodide is a popular red-fluorescent counterstain. Living cells are not permeable to it, but dead cells are (hence acquire dark coloring) and can be detected in the population after exposure treatment of the cells. (**B**) The basis of the MTT assay is that the yellow, water-soluble tetrazole becomes purple, insoluble formazan by the action of mitochondrial dehydrogenase of living cells. Cytotoxic and cytostatic activities can be determined from the optical density of the control and treated cells (ICM: incomplete medium, CM: complete medium). The formazan can be conveniently extracted by DMSO for colorimetric measurement.

Some workers have used staining to reveal the effect of natural compounds on cell viability, mainly the MTT (3-(4,5-dimethylthiazol-2-yl)-2,5-diphenyltetrazolium bromide) assay and the trypan blue exclusion test. However, Wang et al. in 2010 showed that the MTT- and MTS (3-(4,5-dimethylthiazol-2-yl)-5-(3-carboxymethoxyphenyl)-2-(4-sulfophenyl)-2H-tetrazolium)-based assays (Figure 6) underestimate the antiproliferative effect of EGCG [1,61].

The terms “cell viability” or “compound cytotoxicity” have broad meanings in drug discovery. For in vitro monolayer cell cultures, a compound is considered to be cytotoxic if the compound interferes with cellular attachment, or significantly alters cellular morphology, cell growth and cell viability. A variety of assay methods can be used to estimate the number of viable eukaryotic cells after exposure of the investigated compounds. These cell-based assays are often used for screening collections of compounds to determine if the compounds have effects on cell proliferation or show cytotoxic and cytostatic effects. Cell-based assays are also widely used for monitoring organelle function. These screening methods have been devised to examine a broad variety of parameters associated with biochemical events necessary for sustaining viability, especially as evinced by membrane integrity. The quantities emerging from metabolism (especially ATP-based viability) assays are proportional to viable cell number. Cytotoxicity assays determine parameters proportional to the degree of cell death. The fundamental difference between the approaches depends on the length of exposure to the compound (short-term exposures (4 h or less) may adversely affect metabolism markers or ATP content before measurable membrane integrity changes, and long-term exposures (24 h or more), particularly after early primary necrosis, may lead to underestimation of cytotoxicity owing to degradation of marker enzyme activity after its release into the extracellular environment [62,63,64,65].

Most cell viability and cytotoxicity assays can be divided into three categories: those that (i) exploit the loss of membrane integrity; (ii) directly measure metabolic markers or ATP content; and (iii) assess metabolic activity. Other forms of detection exist. Crystal violet staining can reveal the adherence of cells and thus be used to measure the viability of adherent cells [66,67,68]. Determination of the loss of membrane integrity: these assays rely on the breakdown/disintegration of the cell membrane to allow different molecules to enter the cell, or allow intracellular compounds to be secreted to the extracellular area.

Metabolic assays primarily focus on measuring ATP levels, or the reduction of tetrazolium salts, or resazurin dyes inside living cells.

Cellular proliferation causes a change in the ratios of certain metabolites e.g., NADPH/NADP, FADH/FAD, FMNH/FMN, and NADH/NAD. These metabolic intermediates are then capable of reducing tetrazolium salts to formazan product, which can be detected. Resazurin (7-hydroxy-3H-phenoxazin-3-one 10-oxide) is a non-fluorescent redox dye, which when reduced to resorufin becomes a red compound, so the color change can be detected.

The most common method for assaying live cell proliferation is measuring the amount of DNA synthesis, which is done by adding a labeled DNA analog called BrdU (5-bromo-2′-deoxyuridine (BrdU), which is incorporated into the DNA instead of thymidine. To assess the incorporation of BrdU into the DNA colorimetric ELISA or immunohistochemical staining methods are used. A newer approach is to detect the incorporation of the alkyne containing thymidine analog EdU. The incorporation can be detected by a copper catalyzed azide-alkyne cycloaddition.

### 4.2. Limitations and Considerations When Using Membrane Integrity or Metabolic Assays

The listed substrates (Table 4, Table 5, Table 6 and Table 7) all have distinct advantages and disadvantages when compared to each other. Assay sensitivity, noise-to-signal ratio, ease of use, and also reagent stability are all factors that have to be considered. Metabolic assays also need to consider that the reduction of said substrates are impacted by other intracellular metabolic activity, and have no direct effect on the cells viability or cytotoxicity of a studied compound.

In fixed samples such as animal tissues and cell population, proliferation can still be measured, but only by immunostaining for specific proliferative markers. Ki-67 is a nuclear protein that is associated with cell proliferation and ribosomal RNA transcription. Traditional antibodies for Ki-67 can only be used to stain frozen (not paraffin embedded) samples. MIB-1 antibodies however target a different epitope of Ki-67, and thus can be used to stain formalin and paraffin fixed samples, making the use of Ki-67 as a proliferative marker easier. Another commonly used marker is the proliferating cell nuclear antigen. This protein expedites DNA synthesis by holding the polymerase to the DNA, so it is expressed widely in the nucleus during DNA synthesis, making it an effective marker of cell proliferation. Other markers that can be used include MCM2 also. It has to be noted that all such assays measuring DNA synthesis directly or indirectly are sensitive to the stage of the cell in the cell cycle at the time the measurement is carried out.

The most frequently used techniques in cellular adhesion experiments with natural compounds are based on dyes (absorbing or fluorescing) as labels. These methods have several drawbacks; however, (i) in general they are not high-throughput techniques, since the procedures are time-consuming and complicated, (ii) many dyes are cytotoxic and therefore disturb the normal physiological activity of the cells; furthermore their structures are often quite comparable to those of the natural compounds under investigation, leading to interference, (iii) typically only end-point data is gathered; i.e., no information on the kinetics of the processes is obtained, (iv) many natural compounds investigated for possible therapeutic benefits have low molecular weight, and frequently are environmentally sensitive (e.g., to oxidation) and thermally or photolytically unstable. 

A good example of this instability is the green tea polyphenol EGCG [1,50]. Investigating the physiological effects of such compounds by labeling techniques exacerbates the difficulties. 

Recent developments, such as the use of quantum dots (semiconductor nanoparticles) and gold nanoparticles instead of organic dyes as labels do little to alleviate these disadvantages. 

### 4.3. Measurement Techniques for Monitoring Cellular Movement and Adhesion 

Cell migration takes place inside or on the extracellular matrix surface [157]. The lamellae of the cell have to adhere to the matrix components to generate traction forces for cell movement [157]. The actin filaments of the cytoskeleton are linked through the cell membrane to the substrate at focal adhesion points, which are connected by actin filaments such as to promote the direction of movement and are located under the entire body of the cell [157,158]. Hence, adhesion and migration are in close relation to each other; adhesion is essential to achieve cellular movement. Thus, measuring cell migration with migration assays provides information about cell adhesion as well [157]. In Table 8, we summarize the advantages and disadvantages of migration assays. Spreading and adhesion play crucial roles in development and maintenance of cell homeostasis and in many further complex functional processes [159,160]. Many types of assays have been developed to measure the adhesion strengths (forces) and can be categorized into single cell and population cell studies [158,161,162]. Cell adhesion detachment events for single cell studies can be initiated via the breakage of molecular bonds (e.g., optical tweezer techniques and micropipette aspiration), cell population studies via static adhesion (centrifugation technique), and cell population studies via dynamic adhesion (e.g., microfluidic techniques, spinning disk, and flow chamber) [158,161,162]. The measurement of migration and adhesion under experimental in vitro conditions can provide important information about natural compounds, yielding quantitative data about cellular movement and adhesion albeit in a drastically simplified environment compared to the in vivo situation [50].

#### 4.3.1. Label-Free Biosensors

The concept of labeling is rooted in the poor sensitivity of many traditional biological molecular detection techniques. Biomolecules are mostly composed of light elements with small masses and optical polarizabilities and are only rarely fluorescent. The techniques based on electrons, X-rays, visible light and mass spectrometry, which have been so successful in physics and chemistry, become much less potent when moved to the world of biology. By artificially coupling a biomolecule to an artificial entity with an electron density, optical polarizability or mass that may be orders of magnitude greater than that of the biomolecule, the latter suddenly becomes “visible” to the technique being employed to detect it. The euphoria that followed this sudden visibility tended to completely overwhelm continuing appraisal of whether the labeling itself might mask the biological effect that was the primary object of investigation [189].

The advent of new technologies sufficiently sensitive to remove the need to label the biomolecules to make them visible opened up a new world of label-free detection [190,191,192]. Natural compounds usually have small molecular weight where labeling can be problematic or even impossible, especially if their binding pocket is small or embedded. Label-free biosensors are emerging tools to investigate the mode of action of small molecules as well. They eliminate all of the disadvantages of the classical techniques listed above, because these methods do not require labels or dyes, which may disturb the samples. Furthermore, in general, the measurement procedures are not difficult, relatively cheap, and not time-consuming. In the field of natural compound research (for example active compounds in traditional Chinese medicines), novel methods, for example, resonant waveguide grating (RWG) [50,193,194] and holographic microscopy can be applied without using any dyes or other labels [195]. 

These techniques, particularly the highly sensitive waveguide-based methods [57,194,196,197,198,199,200,201,202], constitute highly promising novel phenotypic assays for drug discovery [203] owing to their capacity to reveal the complexity of drug actions and interactions and provide a holistic view of receptor-ligand interactions in living cells. Waveguide sensors are based on the phenomenon of the evanescent field generated by waveguided (usually visible) light being modulated by the presence of drugs, proteins and other biomolecules and living cells [204,205]. An adlayer, which may be constituted from a phospholipid membrane, extracellular matrix proteins or tissue is firstly placed onto the planar waveguide, where it is in contact with physiological medium containing the analyte of interest, such as the natural compound [204,205]. Molecular processes taking place in or on this adlayer modify the parameters of light propagating in the waveguide, any of which can serve as signal transducers [205,206].

Label-free techniques are especially useful for kinetic monitoring of biological events [207,208,209], even at single-cell level [210]. So they have become more and more popular in the field of drug discovery and in other areas focusing on the biological roles of small molecules [1,203]. Furthermore, the same techniques can be readily adapted to measure the dynamics of cell shape changes [211]. Cell adhesion assays and cell migration tests can be achieved in a completely label-free way, with in situ exposure to the natural compounds and, indeed, any drug. For example, a recent study of Peter et al. applied holographic microscopy to quantitatively show that EGCG reduced the motility speed and migration of HeLa cells [195]. Holographic microscopy creates 3D images of cells and the changes in cell morphology due to EGCG could be observed in real time [195].

**Figure 7:**
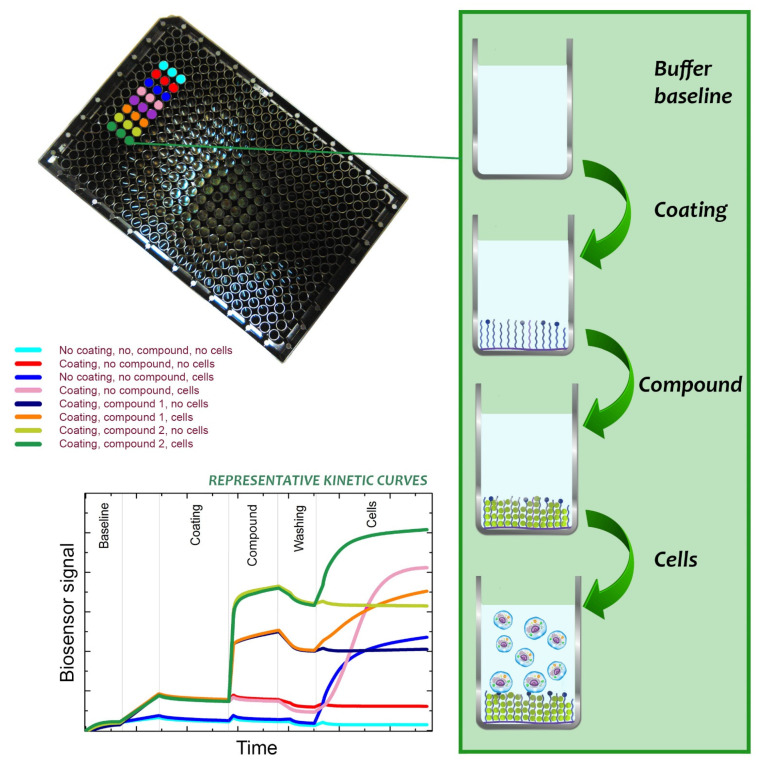
Multicomponent study measuring cancer cell adhesion on a natural compound-treated coating [50]. A 384-well biosensor plate was used in the experiment (**left**) comprising the manipulation steps in a typical well (**right**). Representative kinetic curves are also shown (bottom).

These techniques are more and more important to systematically study the effects of active substances on cell behavior, especially cell adhesion, and to investigate different biological materials (such as membranes and matrices) and living cells in a high-throughput format [50]. In a recent experiment, Peter et al. showed that EGCG can block the RGD (Arg-Gly-Asp) motif, an important cell adhesion ligand and directly affect matrix properties by hydrogen bonding (Figure 7) [50]. The other novelty of this study is that the polymer coating fabrication, its treatment with the small EGCG molecule (a green tea polyphenol), and the observation of cell adhesion could be all studied online using a high-throughput RWG biosensor, with different EGCG and oxidized EGCG concentrations [50]. 

This and similar methodologies should be applicable to other extracellular matrix interactions with small natural compounds (Figure 7). 

Recently, it was concluded that the shape of the kinetic curves obtainable with the label-free biosensor can be used to quantify in vitro cell viability in a fast and highly sensitive manner [59].

Natural compounds are generally small molecules that can self-associate into colloidal aggregates in aqueous buffer [212]. In the early stage of drug discovery, this phenomena is the principal cause of false results [212]. Wang et al. reported RWG-based assays to identify natural compound aggregation and characterize its influence on membrane receptors in living cells [212]. They showed that the colloidal aggregates may cause false activity in DMR desensitization assays [212]. A series of RWG-based assays for colloidal aggregate detection and characterization of their promiscuity were developed [212]. RWG-based assays can be applied as practical tools to distinguish between real and false responses, providing useful reliable results in the early stages of drug discovery [212]. 

#### 4.3.2. Living Cell Movements—Holographic Microscopy

The realization of in situ monitoring of living cell movements (i.e., crawling along the endothelium) is a very important advance [195,213]. There are anyway few techniques to study cell movements; classical ones are mostly directed at migration studies and they also have their drawbacks [195]. For instance, filter assays (Zigmond and Dunn chambers, Boyden chamber) measure cell migration over a membrane in response to chemoattractant compounds [195]. These assays are very specialized, requiring cells to migrate through both a matrix and the pores of a filter [213]; however, very few cell lines can migrate through both of them [195]. Single cell movements can be investigated by using time-lapse imaging, usually requiring fluorescent labels [195,213]. As discussed above, fluorescent imaging may disturb the cells and the imaging time is limited by bleaching of the fluorescent marker [195,213]. In contrast to fluorescent imaging, holographic microscopy is a label-free technique [195,214]. It is usually critical to observe and quantitatively record live cell behavior, especially directional movement (migration) and random movement (motility), and also the shape changes to understand the behavior of the cells in such environments and to be able to make inferences regarding further therapeutic possibilities [195]. Although traditional herbal extracts have become more and more popular for treating illnesses, the literature of systematic quantitative studies is still limited [1]. 

## 5. Preparation of Natural Compounds

Natural products with their broad chemical diversity and bioactivity spectrum are sought after by the pharmaceutical industry and they continue to provide new structures with promising effects and to offer templates for the development of scaffolds of novel drug candidates. However, natural product discovery programs have been abandoned over the past 30 years by many pharmaceutical companies [215,216,217].

Natural products present their own challenges for drug discovery, including screening, isolation, characterization and optimization, which doubtless contributed to the decline in their pursuit by the pharmaceutical industry. In recent years, several technological and scientific developments—such as improved analytical tools, genome mining, engineering strategies, and microbial culturing—are addressing these challenges, preparing the way for new opportunities and interest in natural compounds [218,219].

Here, we briefly summarize recent technological developments that are facilitating natural product-based drug discovery, highlight selected applications and discuss key opportunities. Innovative compound identification from natural products requires a multidisciplinary approach utilizing numerous technologies [220]. 

Isolation and purification of natural compounds are the most important and difficult steps for molecular structure identification, in vitro testing, quantity control and further industrial production. The natural compounds are usually in complex matrix material and the active moieties are present in low concentration [220] which are often accompanied by co-occurring irrelevant common metabolites accumulated in high amounts. Selection of appropriate techniques and strategies is essential for getting the target compounds at high yield. Over the past decades, many types of new extraction, isolation and purification techniques (classical solvent extraction procedures, ultrasound-assisted extraction (UAE), microwave-assisted extraction (MAE), extraction with ionic liquids, accelerated (pressurized) solvent extraction (ASE), supercritical fluid extraction (SFE), extraction on solid phases, distillation methods, membrane filtration, preparative high-performance liquid chromatography (HPLC), counter-current chromatography (CCC), supercritical fluid chromatography (SFC), etc.) have been applied. With developments in separation science and microplate-based in vitro high-throughput screening (HTS) assays, natural compound research gained remarkable momentum recently. Nevertheless, despite these developments of extraction, separation, analytical and structural elucidation techniques, isolation of natural compounds from plants, marine organisms or microorganisms is challenging, with problems including post-harvest changes in material quality [221,222]. Extraction is the first step to separate most natural products from the raw materials. The method progresses through the following stages: (i) solvent penetration into the matrix; (ii) the components dissolve in the solvent; (iii) the components diffuse out of the matrix. A brief but excellent summary of the various extraction methods used for natural products is presented by Zhang and co-workers [223]. A more detailed description is presented in a book chapter by Cavalcanti et al. [224]. The analyses of extracts, fractions, isolates, compounds from different natural matrices, studies on chemotaxonomy, chemical fingerprinting, metabolome elucidation, and structural evaluation have become easier because of the availability of a number of instrumental analytical techniques, e.g., GC-MS, LC-PDA, LC-MS, LC-FTIR, LC-NMR, LC-NMR-MS, and CE-MS, NMR, NIR, FT-IR. 

Atanasov and co-workers [219] have detailed the traditional bioactivity-guided isolation process and its limitations, and also ideas on addressing them. The general process begins with the extraction steps and the method applied determines which type of compounds will be present in the particular extract (solvents with hydrophilic chemical character will result in a higher abundance of hydrophilic compounds). Following the identification of an extract with promising bioactivity, the next step is its (often multiple) consecutive bioactivity-guided fractionation until the individual active compound or compounds are isolated. To isolate such new antiproliferative/antimicrobial compounds from a series of extracts, highthroughput label-free activity screening by label-free biosensors could be applied. The identities of the pure compounds responsible for an extract’s activity can be determined by bioactivity-guided fractionation using novel label-free biosensor systems [1,204,205,206,207,208,209,210,211,212,213,214].

**Figure 8:**
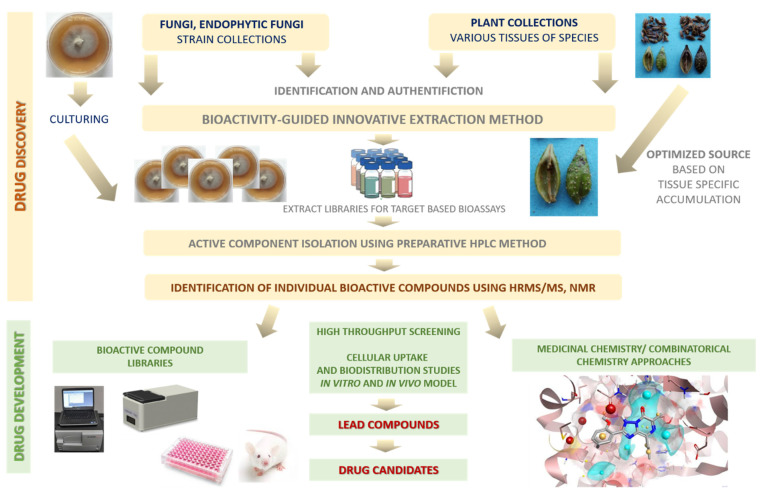
Bioactivity-guided isolation of natural compounds.

A systems biology-guided approach coupled with application of commensurate technologies (genomics, proteomics, metabolomics, in silico modeling, etc.) should provide new opportunities for identifying new and better drug candidates. Molecular libraries of lead compounds from natural products will serve as sources of lead extracts/compounds for innovative drugs (Figure 8).

## 6. Natural Compounds of Plant, Animal and Fungal Origin

We summarize and categorize those natural compounds found in the literature that affect cellular adhesion and migration. The sources of the active compounds are divided into three groups; plants (Table 9), animals (Table 10), or fungi (Table 11). The compounds produced by these organisms include primary and secondary metabolites. They show structural and functional diversities, which are the result of biosynthetic processes modulated by natural selection. Defense molecules—antimicrobial peptides (AMPs)—are commonly produced to resist stresses. These oligo- and polypeptides are synthesized by ribosomes or via non-ribosomal peptide synthetases. Many AMPs exhibit broad-spectrum antimicrobial and anticancer activity. Plants are a remarkably promising source of these molecules and they are also considered to affect plant growth and development [225]. AMPs can be classified into cationic or anionic compounds according to their net charge. Their function, isolation and other aspects have been reviewed [225,226,227,228].

In the cases of plant or fungal origin, it has been well known for centuries that active substances can be extracted from individual plant organs (i.e., leaves, flowers, rhizomes, fruits, seeds, roots, stems, bark and xylem). These parts can be consumed as hot water infusions (e.g., green tea, herbal infusions), as spices (e.g., pepper, clove), as raw fruits or extruded/fermented products (e.g., red wine, olive oil). Such natural compounds are consumed day-by-day as food, but their ability to cure diverse diseases has been known for thousands of years (e.g., in Chinese traditional medicine).

In our tables, if the literature information is too scanty to allow us to be more specific, we simply write P (pathway intervention involving gene expression) or B (cell adhesion inhibition by blocking specific receptor sites) to indicate the mechanism. 

In case of animal origin, the most popular category of substance is serpent venom (Table 10). However, a recent study of Mattia et al. showed that edible insects and invertebrates can be a source of useful polyphenols as well [235]. For example, black ants *(Lasius niger)*, mealworms *(Tenebrio molitor)* and grasshoppers contain the highest levels of total polyphenols [235]. More experiments are needed to understand whether eating insects and other invertebrates might be beneficial to humans [235], although we note that some cultures have, for example, regarded locusts as comestible for millennia.

Statins, cholesterin-lowering active ingredients, are among the most widely prescribed medicines. Naturally occurring statins (e.g., lovastatin and mevastatin) are produced by different fungi, a synthetic derivate (simvastatin) of a fungal fermentation product is found in pharmacopeias alongside synthetic statins (e.g., atorvastatin, fluvastatin). In Table 11, the synthetic derivatives are labeled (syn). Lovastatin is naturally produced by e.g., *Pleurotus ostreatus* (oyster mushroom) [236] and mevastatin was first isolated from the *Penicillium citrinum* [237]. Several pleiotropic effects of statins have been revealed, including enigmatic effects on cancer [238,239,240]. Naturally occurring statins can suppress cell migration, invasion, and cell adhesion (Table 8). The effects of such suppression on cancer metastasis would appear to be obvious. We focus on studies in which cell migration, invasion and adhesion have been studied in vitro. Another metabolite type, the macrosphelides (MSs; A, B, C, D, E, F, G, H, etc.), are macrolides produced by several fungal strains, for example *Microsphaeropsis* sp. [241]. These compounds and metabolites from the peribysin group are also detailed in Table 11.

**Table 11:** Effects of fungi-derived natural compounds on cell adhesion and movement. The table summarizes the applied methods and techniques as well.

Active Substance	IUPACName (CAS Number)	Source	Cellular Effect	Effect Type	Molecular Mechanisms	Tested Cell Line ^1^, Animal	Method	Ref.
*Ganoderma lucidum* polysaccharide		*Ganoderma lucidum*	Inhibition of tumour cell adhesion	P	Ateration in β1-integrin expression	MT-1	Coomassie blue staining, Western blot	Wu et al. 2006 [47]
Rhodostomin peptide 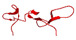		*Pichia pastoris*	Inhibition of cell adhesion, platelet aggregation and the binding of fibrinogen to platelet by ARGDWN mutants	B	Prevention of integrin αIIbβ3 interaction	CHO K562	Mass spectrometry, Fibrinogen binding assay, Flow cytometry, Platelet aggregation assay, Nuclear magnetic resonance spectroscopy, Molecular docking	Chang et al. 2017 [56]
Cyclopeptolide HUN-7293 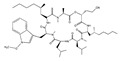	3-((2R,5S,8S,11S,14S,17R,20S)-8,11-diisobutyl-14-((1-methoxy-1H-indol-3-yl)methyl)-7,13,19,20-tetramethyl-18-methylene-5-((R)-2-methylheptyl)-17-((R)-2-methylhexyl)-3,6,9,12,15,21-hexaoxo-1-oxa-4,7,10,13,16,19-hexaazacyclohenicosan-2-yl)propanenitrile (129893-84-1)	*Bartalinia robillardoides*	Inhibition of VCAM-1 and ICAM-1	P	Inhibition of the expression of VCAM-1 and ICAM-1	HMEC-1	ELISA	Schreiner et al. 2004 [242]
Cytochalasin-E 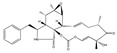	(1E,4S,6R,7E,11aS,14S,14aS,15S,15aR,16aR,16bS)-14-benzyl-6-hydroxy-4,6,15,15a-tetramethyl-3,13,14,14a,15,15a,16a,16b-octahydro-[1,3]dioxacyclotridecino[4,5-d]oxireno[2,3-f]isoindole-5,10,12(4H,6H)-trione (36011-19-5)	*Mycotypha* sp. UMF-006 (soil)	Inhibition of cell aggregation and adhesion	No data	No data	HL-60 CHO	Cell aggregation assay, Adhesion assay (CFSE-labelling	Takamatsu et al.n 2002 [243]
Cordyceptin 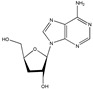	(2R,5S)-2-(6-amino-9H-purin-9-yl)-5-(hydroxymethyl)tetrahydrofuran-3-ol (73-03-0)	*Cordiceps militaris*	Inhibition of motility, invasion and migration	P	Inhibition of PI3K/Akt pathway and expression of claudin family proteins. Downregulation of MMP activity	LNCaP	Wound healing migration assay, MTT assay, Boyden chamber, Matrigel invasion assay, Measurement of TER (EVOM voltohmmeter)	Jeong et al. 2012 [244]
Fusarisetin A 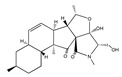	(3S,3aR,5S,5aS,5bS,7aS,9R,11aR,11bS,12aR)-3a-hydroxy-3-(hydroxymethyl)-2,5,9,11b-tetramethyl-3,3a,5,5a,5b,7a,8,9,10,11,11a,11b-dodecahydro-12H-benzo[4′,5′]indeno[2′,1′:3,4]furo[2,3-c]pyrrole-1,12(2H)-dione (1300041-53-5)	*Fusarium* sp. FN080326	Inhibition of migration	P	Pathway inhibition	MDA-MB-231	Scratch wound assay, Boyden chamber transwell assay	Xu et al. 2012 [245]
Terrein 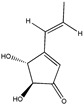	(4R,5S)-4,5-dihydroxy-3-((E)-prop-1-en-1-yl)cyclopent-2-en-1-one (582-46-7)	*Aspergillus terreus* CRI301	Inhibition of adhesion and migration	P	Downregulation of MMP-2 and MMP-9 transcription. Reduction of protein levels for the Rho GTPases	MDA-MB-231 MCF-7	Cell adhesion assay (crystal violet staining), Wound healing assay, Matrigel cel migration assay	Kasorn et al. 2018 [246]
Ophiobolin A 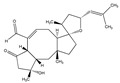	(3S,3aR,3′S,5′R,6aS,9R,9aS,10aR,E)-9-hydroxy-3′,9,10a-trimethyl-5′-(2-methylprop-1-en-1-yl)-7-oxo-1,3a,4,4′,5′,6a,7,8,9,9a,10,10a-dodecahydro-2H,3′H-spiro[dicyclopenta[a,d][8]annulene-3,2′furan]-6-carbaldehyde (4611-05-6)	*Drechslera gigantea*	Decreased the 2D-migration potential	No data	No data	U373 MG Glioblastoma multiforme (GBM)	MTT assay, Quantitative video microscopy, Immunofluorescence/pseudo-confocal microscopy, Flow cytometry	Bury et al. 2013 [247]
Fusicoccin A 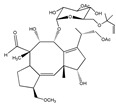	(2S)-2-((1S,4R,5S,6S,9S,10aR,E)-4-(((2S,3R,4S,5R,6R)-4-acetoxy-3,5-dihydroxy-6-(((2-methylbut-3-en-2-yl)oxy)methyl)tetrahydro-2H-pyran-2-yl)oxy)-6-formyl-1,5-dihydroxy-9-(methoxymethyl)-6,10a-dimethyl-1,2,4,5,6,6a,7,8,9,10a-decahydrodicyclopenta[a,d][8]annulen-3-yl)propyl acetate (20108-30-9)	*Fusicoccum amygdali*	Decreased the 2D and 3D-migration potential. Decreased adhesion	No data	No data	U373-MG Glioblastoma multiforme (GBM)	Quantitative video microscopy, Boyden chamber assay, In vitro adhesion assay (hematoxylin staining)	Bury et al. 2013 [248]
Altersolanol A 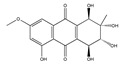	(1R,2S,3R,4S)-1,2,3,4,5-pentahydroxy-7-methoxy-2-methyl-1,2,3,4-tetrahydroanthracene-9,10-dione (22268-16-2)	*Stemphylium globuliferum*	Inhibition of migration	P	Inhibition of TNFα-activated NF-κB signalling pathway. Inhibition of NF-κB-mediated MMP expression	A549	Incucyte Live-Cell imaging system, Videomicroscopy analysis, Scratch wound assay, flow cytometry, Western blot	Teiten et al. 2013 [249]
Sphaeropsidin A 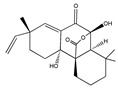	(2R,4aR,4bR,8aS,9S)-4a,9-dihydroxy-2,8,8-trimethyl-2-vinyl-2,3,4,4a,5,6,7,8,8a,9-decahydro-10H-9,4b-(epoxymethano)phenanthrene-10,12-dione (38991-80-9)	*Smardea* sp. AZ0432	Inhibition of migration	No data	No data	MDA-MB-231	Wound healing assay	Wang et al. 2011 [250]
Gliotoxin 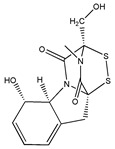	(3R,5aS,6S,10aR)-6-hydroxy-3-(hydroxymethyl)-2-methyl-2,3,5a,6-tetrahydro-10H-3,10a-epidithiopyrazino[1,2-a]indole-1,4-dione (67-99-2)	Unidentified fungal strains Y90086/Y80805	Inhibition of migration	No data	No data	HUVEC	Wound migration assay	Lee et al. 2001 [251]
Methylthiogliotoxin 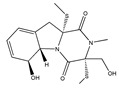	(3R,5aS,6S,10aR)-6-hydroxy-3-(hydroxymethyl)-2-methyl-3,10a-bis(methylthio)-2,3,5a,6,10,10a-hexahydropyrazino[1,2-a]indole-1,4-dione (74149-38-5)							
Cytochalasin D 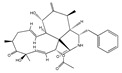	(3S,3aR,4S,6S,6aR,7E,10S,12R,13E,15R,15aS)-3-benzyl-6,12-dihydroxy-4,10,12-trimethyl-5-methylene-1,11-dioxo-2,3,3a,4,5,6,6a,9,10,11,12,15-dodecahydro-1H-cycloundeca[d]isoindol-15-yl acetate (22144-77-0)	Not mentioned (but many fungi produce, e.g., *Zygosporium mansonii)*	Inhibition of actin polymerization, decreased cell motility and colonization. Inhibition of actin polymerization	No data	No data	MFC-7 A549	Quantitative videomicroscopy, Scratch wound assay	Hayot et al. 2006 [252]
FTY720 (fingolimod) 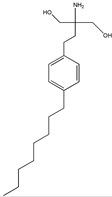	2-amino-2-(4-octylphenethyl)propane-1,3-diol (162359-55-9)	*Isaria sinclairii*	Inhibition of cell migration, motility and invasion	P	Inhibition of RhoA-GTPase expression	DU145 PC3	Wound closure assay, 3D collagen colony forming assay	Zhou et al. 2006 [253]
Staitins								
Lovastatin 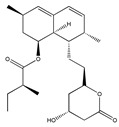 * (Fluvastatin (syn)) 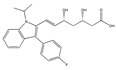	(1S,3R,7S,8S,8aR)-8-(2-((2S,4R)-4-hydroxy-6-oxotetrahydro-2H-pyran-2-yl)ethyl)-3,7-dimethyl-1,2,3,7,8,8a-hexahydronaphthalen-1-yl (S)-2-methylbutanoate (75330-75-5)	Pleurotus ostreatus **	Inhibition of EGF-induced migration and invasiveness	P	Inhibition of translocation of RhoA	PANC-1	Matrigel invasion assay	Kusama et al. 2001 [254]
	(3S,5R,E)-7-(3-(4-fluorophenyl)-1-isopropyl-1H-indol-2-yl)-3,5-dihydroxyhept-6-enoic acid (93957-54-1)		Inhibition of tumor cell attachement and migration	No data	No data	F3II	Adhesion assay, Migration assay (in vitro wound assay)	Alonso et al., 1998 [255]
			Suppressed the EGF-induced cell adhesion, actin filament reorganization and transmigration	P	Suppression of Rho/ROCK and FAK/paxillin signaling	ARO	Matrigel invasion assay, Cell adhesion assay	Zhong et al., 2005 [256]
			Inhibition of adhesion. Reduced tumor cell migration, attachement and motility. Changed the tumor cell shape	P	Reduced membrane localization of Rho protein	F3II	Adhesion assay, Migration assay (in vitro wound assay)	Farina et al., 2002 [257]
			Reduced migration and invasion	P	Diminished ERK signaling. Impaired the regulation of the mevalonate- and the Ras-Raf-MEK-ERK pathway. Affected the post-translational modification of H-Ras and Rac1	U87 U343	Migration assay, Matrigel invasion assay	Afshordel et al., 2014 [258]
Lovastatin 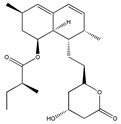	(1S,7S,8S,8aR)-8-(2-((2R,4R)-4-hydroxy-6-oxotetrahydro-2H-pyran-2-yl)ethyl)-7-methyl-1,2,3,7,8,8a-hexahydronaphthalen-1-yl (S)-2-methylbutanoate (73573-88-3)	Pleurotus ostreatus **	Inhibition of invasion and migration	P	Alteration in expression of matrix-metalloproteases	M14	Matrigel invasion assay, Integrin-mediated binding assays	Glynn et al., 2008 [259]
Mevastatin 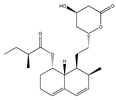	(1S,7S,8S,8aR)-8-(2-((2R,4R)-4-hydroxy-6-oxotetrahydro-2H-pyran-2-yl)ethyl)-7-methyl-1,2,3,7,8,8a-hexahydronaphthalen-1-yl (S)-2-methylbutanoate (73573-88-3)	Penicillium citrinum **				HT144		
Simvastain (syn) 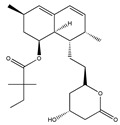	(1S,3R,7S,8S,8aR)-8-(2-((2S,4R)-4-hydroxy-6-oxotetrahydro-2H-pyran-2-yl)ethyl)-3,7-dimethyl-1,2,3,7,8,8a-hexahydronaphthalen-1-yl 2,2-dimethylbutanoate (79902-63-9)	*Aspergillus terreus*				SK-MEL-28		
Macrosphelides (MSs)								
Macrosphelide A 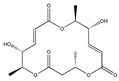	(4S,7E,9R,10S,13E,15R,16S)-9,15-dihydroxy-4,10,16-trimethyl-1,5,11-trioxacyclohexadeca-7,13-diene-2,6,12-trione (172923-77-2)	*Microsphaeropsis* sp. FO-5050 (soil)	Inhibition of HL-60 cell adhesion to HUVEC	B	Blocking the binding of SLex to ELAM-1	HL-60	Cell adhesion assay	Hayashi et al., 1995 [260]
B 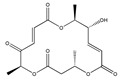	(4S,7E,9R,10S,13E,16S)-9-hydroxy-4,10,16-trimethyl-1,5,11-trioxacyclohexadeca-7,13-diene-2,6,12,15-tetraone (172923-78-3)					HUVEC		
Macrosphelide C 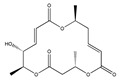	(4S,7E,10S,13E,15R,16S)-15-hydroxy-4,10,16-trimethyl-1,5,11-trioxacyclohexadeca-7,13-diene-2,6,12-trione (199731-56-1)	*Microsphaeropsis* sp. FO-5050 (soil)	Inhibition of adhesion	No data	Not discussed	HL-60	Cell adhesion assay	Takamatsu et al., 1997 [261]
D 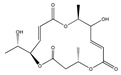	(2S,6R,7E,11S,13E)-12-hydroxy-6-((S)-1-hydroxyethyl)-2,11-dimethyl-1,5,10-trioxacyclopentadeca-7,13-diene-4,9,15-trione (199731-57-2)					HUVEC		
Macrosphelide J 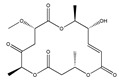	(4S,9R,10S,13S,16S,E)-9-hydroxy-13-methoxy-4,10,16-trimethyl-1,5,11-trioxacyclohexadec-7-ene-2,6,12,15-tetraone (239094-07-6)	*Microsphaeropsis* sp. FO-5050 (soil)	No effect	No effect	No effect	HL-60 HUVEC	Cell adhesion assay	Fukami et al., 1999 [262]
K 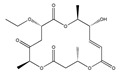	(4S,9R,10S,13S,16S,E)-13-ethoxy-9-hydroxy-4,10,16-trimethyl-1,5,11-trioxacyclohexadec-7-ene-2,6,12,15-tetraone							
epi-5’-hydroxymycosporulone 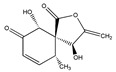	(4S,5R,6R,10R)-4,6-dihydroxy-10-methyl-3-methylene-2-oxaspiro[4.5]dec-8-ene-1,7-dione (238735-98-3)							
Macroshelide A 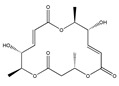 and structurally related compounds of Macroshelide A (Macroshelide C, E, F, G, H, I, L and M)	(4S,7E,9R,10S,13E,15R,16S)-9,15-dihydroxy-4,10,16-trimethyl-1,5,11-trioxacyclohexadeca-7,13-diene-2,6,12-trione (172923-77-2)	*Periconia byssoides* OUPS-N133	Inhibition of HL-60 cell adhesion to HUVEC	No data	No data	HL-60 HUVEC	Cell adhesion assay with MTT staining (“Miki’s method” [263])	Yamada et al., 2001, 2002, 2007 [264,265,266]
Macroshelide B 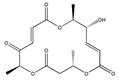	(4S,7E,9R,10S,13E,16S)-9-hydroxy-4,10,16-trimethyl-1,5,11-trioxacyclohexadeca-7,13-diene-2,6,12,15-tetraone (172923-78-3)	*Microsphaeropsis* sp. FO-5050 (soil)	Inhibition of cell binding to HUVEC	B, P	Blocking the binding of sLex to E-selectin. Suppressed the expression of adhesion molecules.	B16/BL6 L5178Y-ML HUVEC	Adhesion assay (CFSE-labelling), flow cytometry analysis	Fukami et al., 2002 [267]
Peribysins								
Peribysin A 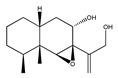 and structurally related compounds of Peribysin A (Peribysin B, C, D, E, F, G, H, I and J )	(1aR,2S,3aR,7S,7aR,7bS)-1a-(3-hydroxyprop-1-en-2-yl)-7,7a-dimethyldecahydronaphtho[1,2-b]oxiren-2-ol	*Periconia byssoides* OUPS-N133	Inhibition of HL-60 cell adhesion to HUVEC	No data	No data	HL-60 HUVEC	Cell adhesion assay with MTT staining (“Miki’s method” [263])	Yamada et al., 2004, 2005, 2006, 2007 [266,268,269,270]

^1^ MT-1: human breast malignant carcinoma, CHO: Chinese hamster ovary cells, K562: human immortalized myelogenous leukemia cells, HMEC-1:human dermal microvascular endothelial cells, HL-60: human leukemia cells, LNCaP: androgen-sensitive human prostate adenocarcinoma, MDA-MB-231: human breast adenocarcinoma, MCF-7: human breast adenocarcinoma, U373-MG: human glioblastoma astrocytoma, A549: human lung epithelial cells, HUVEC: human umbilical vein endothelial cells, DU145:human prostate cancer cells, PC3: human prostate cancer cells, PANC-1: human pancreatic cancer cells, F3II: sarcomatoid mammary carcinoma, ARO: anaplastic thyroid cancer cells, U87: human primary glioblastoma cells, U343: glioblastoma cells, M14: human melanoma cells, HT144: human melanoma cells, SK-MEL-28:human melanoma cells, B16/BL6: mouse melanoma cells, L5178Y-ML: mouse lymphoma cells. * Fluvastatin is a synthetic derivative of lovastatin. Experimental results of fluvastatin are only related to the work of Kusama et al. [254] ** In general, the cited publications do not detail the source of statins. In this table, the typical natural sources of these compounds are given; *Pleurotus ostreatus* and *Penicillium citrinum*. In the case of lovastatin, *Aspergillus terreus* is a main source as well.

## 7. SARS-CoV-2 and Possible Treatments with Herbal Extracts

**Figure 9:**
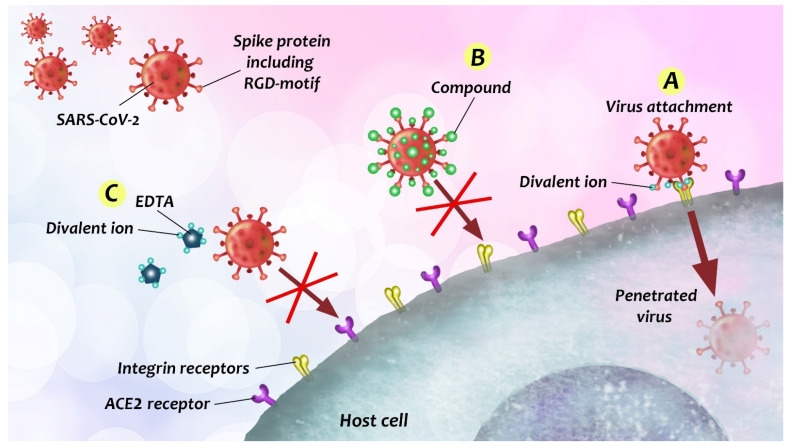
Schematic illustration of SARS-CoV-2 attachment and two possible examples of ways to inhibit this process. (**A**) Virus can attach to ACE2 receptor or integrins of the host cell. RGD-integrin interaction occurs in calcium-dependent manner [274]. As the result of the process, the virus penetrates into the cell and starts to copy itself. (**B**) Several compounds bind to the spike protein or even may alter it [275] and prevent virus-receptor attachment. (**C**) Lowering divalent ion concentrations in the lungs with pulmonary EDTA chelation therapy may inhibit virus-host interaction [274].

A novel coronavirus (nCoV) started infecting humans since late 2019. The pathogen is called “Severe Acute Respiratory Syndrome-related Coronavirus 2” (SARS-CoV-2). It can cause a fatal respiratory disease, called Coronavirus disease 2019 (COVID-19), and acute respiratory distress syndrome (ARDS) as well [271]. The COVID-19 pandemic spread quickly inducing a worldwide problem, because the virus is highly contagious; transmission occurs presumably mainly via airborne droplets [271]. SARS-CoV-2 belongs to the genus *Betacoronavirus* of the large family of *Coronaviridae* [271]. Externally, the virus displays a corona-shaped layer of spikes that play a significant role in the infection process [272]. The virions are able to recover from the drastic mechanical perturbation imposed by atomic force microscopy [272]. The global structure is temperature-resistant, but the virion surface is denuded of spikes if heated [272]. These properties obviously influence the infectivity of the virus. The spike protein is a large glycoprotein trimer, which contributes to host receptor binding, cell tropism and pathogenesis [271]. A recent study showed that the surface of the native virion displays a dynamic brush owing to the rapid motions and flexibility of the spikes [272]. By binding host receptors, the virus genome penetrates into the cytoplasm of the host cell [271]. The angiotensin-converting enzyme II (ACE2) is a receptor for SARS-CoV-2, but it is suggested that the virus may use integrins too as receptors, binding to them through the conserved RGD motif [50,51,273]) present on the spike protein [271]. Members of the integrin family are commonly used as receptors by many other human viruses as well, and RGD is the minimal sequence required for binding [271]. Integrins have high expression in lungs and other vital organs whereas ACE2 is found to have negligible occurrence in the lungs [274]. Expression of integrins is high in lung cells (especially αVβ6, αVβ8, α5β1) and the ICAM-1. Thus, the high infectivity of the virus may be at least partly due to the RGD-integrin-mediated cell-adhesive property [274]. Phosphorylation sites on the spike protein induce Tyr, PKC and cAMP signaling pathways, which activate calcium ion channels or get activated by calcium [274]. Thus the RGD-integrin interaction clearly occurs in a calcium-dependent manner [274]. This interaction may then unleash a “cytokine storm” due to TNFα and IL-6 activation [274]. The lowering of divalent ion concentrations in the lungs by pulmonary EDTA chelation therapy may inhibit virus-host attachment [274]. Glycans may have multiple roles during viral entry [275]. Inhibition of N-glycan biosynthesis was shown to enhance spike protein proteolysis, leading to a decrease of receptor-binding domain presentation on the virus [275]. Thus, another idea for treatment is to administer chemical inhibitors of glycosylation [275]. Hence, although vaccines have already been developed and authorized for emergency use, extensive research work and brainstorming continue for the development of future drugs against SARS-CoV-2 (Figure 9).

Already-existing synthetic drugs (favipiravir, ivermectin, remdesivir, among others) have been shown to be effective for treatment, but the application of these drugs may have direct or indirect side effects (for example, pain, liver problems, allergy, etc.) [276]. Plants are an abundant source of natural antiviral compounds that may be an effective option, because most of them are safer compared to synthetic drugs, although there are exceptions [276]. Some of these metabolites have protective effects against different microbes; however, the role of most metabolites still remains unknown for us. Bhuiyan et al. created a large collection of antiviral compounds from 219 medicinal plants [276], from which, it can be inferred that polyphenols work against coronaviruses by actuating or inhibiting cellular signaling pathways or inhibiting 3-chymotripsin-like protease (3CL^pro^) and papain-like protease (PL^pro^) [276]. Polyphenol compounds from *Broussonetia papyrifera*, *Sambucus* and *Pelargonium*, and flavonoid-type compounds (quercetin, apigenin) showed activity against human coronavirus [276,277]. Different types of alkaloids (for example tylophorine, 7-methoxy cryptopleurine, etc.) have anti-SARS activity by inhibiting protease and RNA and protein synthesis, and chloroquine has been reported to have anti-SARS-CoV-2 activity [276]. Saponins (amphipatic triterpenes) showed antiviral effects against a lot of viruses (*Influenza* virus, *Dengue* virus, rotaviruses, among others) [276,278]. Among triterpenes, ginkgolide A can strongly inhibit the SARS-CoV-2 protease [276], and glycyrrhizin was found to be effective against influenza [278]. In the case of SARS-CoV-2, glycyrrhizin was found to have the highest binding affinity with the viral S protein [276,279], but curcumin, apigenin and chrisophanol also bind to this part of the virus according to in silico molecular docking [279]. In a recent study, EGCG from green tea beverage was shown to inhibit infection of live virus and its variants by inhibiting spike binding to ACE2 receptor [280].

It should be noted that the risk factors for a severe course of COVID-19 in intensive care unit patients are chronic obstructive pulmonary disease, renal dysfunction, hypertension, diabetes mellitus and coronary heart disease. Elderly adults and patients with chronic illnesses and obesity are vulnerable [278]. There is preliminary evidence that nutrient-related disorders are associated with greater susceptibility to infection [278].

Herbal remedies have a potentially preventive effect, mainly acting through supporting the immune system, for example *Astragalus membranaceus* or *Echinacea purpurea* [277]. Clinical studies showed that extracts from *Pelargonium sidoides*, *Sambucus nigra* and *Cistus incanus* are effective treatments of infectious respiratory illnesses [277,278]. Kalus et al., a decade before the COVID-19 pandemic, showed that *Cistus incanus* extract significantly decreased the symptoms of 160 patients with infections of the upper respiratory tract (caused by bacteria, influenza, and other viruses) and the level of C-reactive protein inflammatory marker was also decreased [281]. EGCG green tea polyphenol was shown to bind to the hemagglutinin of influenza virus [281] as well, implying that regular consumption of green tea should decrease the influenza infection rate, too [278]. In summary, consuming extracts of herbs, vegetables and fruits improve overall health due to their phytochemicals and nutrients, and these compounds may prevent or attenuate the symptoms of COVID-19.

## 8. Conclusions

Most investigations into the effect of natural products on cellular adhesion are focused on the adhesion molecules belonging to the immunoglobulin superfamily of CAMs (ICAM-1 and VCAM-1), and selectins and integrins [5]. These molecules are implicated in several widespread diseases, such as various types of inflammation, rheumatism, atherosclerosis and cancer. Metabolites obtained from plants, fungi and venoms may offer therapeutic potential by regulating adhesion molecules, generally down-regulation [5]. We summarized natural metabolites having diverse structures, which influence cellular adhesion and migration by modulation of adhesion molecules. The reported effects of natural products, either presented to model cell systems as complex extracts or as purified active principles, generally manifest themselves as: (i) inhibition of cell-cell adhesion (i.e., circulating to endothelial cells); and (ii) a general anti-inflammatory effect. The mechanism of (i) is either the blocking of specific adhesion sites (such as the RGD motif and/or its binding complement) on the cell surface (the group of mechanisms labeled B—blocking adhesion receptors on the surface of immune or tissue cells, or motifs in the ECM), or the downregulation of the cell adhesion molecules (the group of mechanisms labeled P—inhibiting the dephosphorylation, translocation or binding of certain factors). The latter group is often effected by inhibition of signaling pathways involving NF-κB. The mechanism of (ii) is by suppressing ROS, achieved by the sacrificial oxidation of the natural product. To map the properties and effects of natural compounds, “classical” labeling techniques have been applied to monitor cellular adhesion and movement. New label-free methods can provide more information (especially kinetic information) about the effects of extracts more sensitively and conveniently without using any cell physiology-altering dyes or other labels. In this review, the preparation method of natural compounds was summarized and the natural product banks were mentioned as well. We systematized recent studies about natural compounds referring to their effects on cell adhesion and movement; the active substances were categorized based on their origin (floral, faunal or fungal). From these collected results it can be clearly seen that applying natural compounds can be a cure against tumors and inflammatory diseases, but more clinical tests would be desirable. Synthetically modified versions of the substances can be also used to cure illness. Finding new, still unknown natural compounds and to map the exact effects of already known extracts from traditional medicine are both important to create new, better, and more effective therapeutic drugs. Some plant extracts showed antiviral activity on SARS-CoV-2 as well, thus natural compounds may be used to attenuate or even prevent the symptoms of COVID-19 as well.

Our review aimed at collecting evidence in order to answer a specific research question [282], namely the role of natural compounds in cellular adhesion and migration, including the characterization methods of these bioactive compounds. We have elucidated the main points of interest (definition of natural compounds and their isolation, adhesion and migration process, effects on cell viability, invasive and non-invasive assays, label free techniques) with inclusion and exclusion criteria. We have made a careful and systematic search of the literature, using the following keywords: natural compound of plant, fungi and other origin, cell adhesion, migration, motility, movement, CAM, integrin, cancer cell, stimulation, inflammation, viability, cytotoxicity, flow cytometry, dyes, label-free, biosensors, preparation, isolation, intracellular pathogens SARS-CoV-2. Approximately 280 relevant paper wereselected. Very recent (2021) and old references (articles from the 1970s and even from 1962) were evaluated. Our criteria were that the compound must be a natural one (i.e., natural origin), with effect on cell adhesion and/or migration.

## Figures and Tables

**Table 1:** Ligands and functions of different integrins of human leukocytes (RGD, Arg-Gly-Asp sequence; VCAM-1, vascular cellular adhesion molecule-1; ICAM-1, Intercellular adhesion molecule-1) [10,30,31,32,33].

Integrin	Ligands	Functions
β1	RGD, VCAM-1, E-cadherin	Adhesion
β2	iC3b, fibrinogen, ICAM-1	Adhesion, phagocytosis, apoptotic cell clearance
β6	RGD, fibronectin	Adhesion, endocytosis, inflammation
β7	RGD, VCAM-1	Adhesion, inflammation

**Table 2:** Different modes of pathway intervention involving gene expression by natural compounds.

Mode	Action
P1	Inhibiting the dephosphorylation of I κB and, hence, the activation of NF-κB
P2	Inhibiting translocation of activated NF-κB into the nucleus
P3	Inhibition of binding of activated NF-κB to promoter sites for CAM expression

**Table 3:** Three types of mechanisms inhibiting cell adhesion by blocking specific receptor sites.

Mechanism	Action
B1	Blocking adhesion receptors on the surface of mobile cells (e.g., leukocytes)
B2	Blocking adhesion receptors on the surface of tissue cells (e.g., of the endothelium)
B3	Blocking adhesion motifs in the extracellular matrix (ECM)

**Table 4:** Absorbance-based and colorimetric cell viability and cytotoxicity assays ^1^.

Cell Viability and Cytotoxicity Assays Absorbance-Based or Colorimetric					
		Principal	Advantage	Disadvantage	References
Dye exclusion assays	Trypan blue	membrane integrity	simple, easy to use, economic, fast	hemocytometer required, counting error, difficult to process large number of samples, cannot be distinguished healthy cells and cell function lost alive cells, toxic on mammalian cells	Jonston 2010 [69], Strober 2001 [70], Aslantürk and Celik 2013 [71], Stone et al., 2009 [72], Yip and Auersperg 1972 [73], Ruben 1988 [74]
	Eosin (Erythrosin B)	membrane integrity	economic, versatility, biosafety	time-consuming, labor-intensive, sensitive for the contamination, filling rate, and inter-user variation	Kim et al., 2016 [75], Marmion 1979 [76]
Colorymetric assays	MTT	enzyme (mitochondrial) activity	easy to use, safe, high reproductibility, widly used	organic solvents required, significant well-to-well error, interaction with compounds results false positive, or false negative data	Stone et al., 2009 [72], Aslantürk et al. [77], Bopp and Lettieri 2008 [78], Mosmann 1983 [79]
	MTS		easy to use, rapid, sensitive, economic, specific, inexpensive	measured absorbance level is influenced by incubation time, cell type, and cell number; optimal incubation time: 1–3 h	Berg et al., 1994 [80], Tominaga et al., 1999 [81], Rotter et al., 1993 [82], Buttke et al., 1993 [83], Promega Technical Bulletin [84], Cory et al., 1991 [85], Riss et al., 1992 [86]
	XTT		results water soluble crystals, quick, sensitive, easy-to-use, safe; highly sensitive and accurate	strongly depends on reductive capacity of viable cells due to pH, cellular ion concentration, cell cycle variation, environmental factors	Scudiero et al.1988 [87]
	WST-1		easy to use, high reproducibility, wildly used, no interference with phenol red, water soluble dye, no additional incubation time	relatively long incubation time (2 h)	Ishiyama et al., 1993 [88]
	WST-8		not cell permeable; cells can be used after the assay; water soluble formazan	intracellular metabolic activity can influence the reduction of WST-8	Tominaga et al., 1999 [81], Strober 2001 [70]
	LDH (lactate dehydrogenase)	enzyme (lactate dehydrogenase) activity	reliability; quick, simple evaluation, indicator of cell death	serum incompetence	Decker and Lohmann-Matthes 1988 [89], Schins et al., 2002 [90], Fotakis et al., 2006 [91], Lappalanien et al., 1994 [92]
	SRB (sulforhodamine B)	SRB-protein interaction	simple, fast, sensitive, good linearity with cell number, metabolism independent, high reproducibility	homogenous cell suspension is required; cellular clumps and aggregates interfere with SRB	Skehan et al., 1990 [93]
	NRU (neutral red uptake)	neutral red uptake and lysosomal accumulation	good marker for lysosomal damage, fast and simple evaluation	influenced by pollutants	Borenfreund and Puerner 1984 [94], Repetto et al., 2008 [95], Ringwood et al.1998 [96]
	Crystal violet	binding for proteins and DNA of viable cells	quick; chemical inhibtors can be incorporated into the assay	metabolism affected compounds can not be tested, not able to measure cell proliferation rate	Feoktisova et al., 2016 [66], Geserick et al., 2009 [97], Degterev et al., 2008 [98], Sun et al., 2012 [99], Feoktisova et al., 2011 [100]

^1^ Trypan Blue: Staining with trypan blue is one of the oldest viability assays. In a viable cell, the intact membrane will prevent trypan blue from entering cells. In dead or dying cells, trypan blue will enter the cell, staining it blue. This method was traditionally quantified manually using microscopes and hemocytometers, making it very labor-intensive. However, the recent availability of affordable automated cell counters makes this assay less time consuming and more accurate. MTT (3-(4,5-dimethylthiazole-2-yl)-2,5-diphenyl-tetrazolium bromide) is a tetrazolium salt that gets reduced by both mitochondrial and extra-mitochondrial dehydrogenases to form insoluble blue formazan crystals, meaning a solubilization step is required before the assay can be read. MTS/XTT: MTS (3-(4,5-dimethylthiazol-2-yl)-5-(3-carboxymethoxyphenyl)-2-(4-sulphophenyl)-2H-tetrazolium) and XTT (2,3-bis-(2-methoxy-4-nitro-5-sulfophenyl)-2H-tetrazolium-5-carboxanilide) substrates are similar to MTT. However, one advantage is that the reactions are carried out intracellularly in the presence of the intermediate electron acceptor phenazine methosulfate (PMS), which enhances their sensitivity. In addition, the reduced formazan product is soluble and gets released to the culture media, removing the need for the extra solubility step that is required with MTT. However, phenol red in cell culture media, fatty acids, and serum albumin have all been reported to distort data obtained from MTS, XTT, and WST assays over prolonged incubation periods. WST: Water-soluble tetrazolium salts (WSTs) are cell-impermeable tetrazolium dyes that get reduced extracellularly via plasma membrane electron transport, and combined with the electron acceptor PMS to generate water-soluble formazan dyes. LDH assay: Lactate dehydrogenase is a ubiquitous, stable cytoplasmic enzyme that converts lactate to pyruvate. If the cell membrane has been damaged, LDH, and therefore, its enzymatic activity is released from cells and can be detected in cell culture media.

**Table 5:** Fluorometric cell viability and cytotoxicity assays ^1^.

Fluorometric Assay				
	Principal	Advantage	Disadvantage	References
AlamarBlue (resazurin reduction assay)	enzyme (mitochondrial and other (e.g., *Diaphorases*)) activity	relatively inexpensive, sensitive, multiplexed with other methods (e.g., Caspase activity)	fluorescence interference with tested compound; direct cytotoxic effect can be occured (depending on incubation time)	O’Brien et al., 2000 [101], Ahmed et al., 1994 [102], Page et al., 1993 [103], Markossian et al., 2004 [65], Pace et al., 2013 [104]
CFDA-AM (5-carboxyfluorescein diacetate, acetoxymethyl ester)	plasma membrane integrity	can be used parallel with alamarBlue on the same set of cells	fluorescence interference with tested compound	Bopp et al., 2006 [103], Schreer et al., 2005 [105], Ganassi 2000 [106]
protease viability marker assay; GF-AFC assay (glycylphenyl-alaninyl)-aminofluoro-cumarine)	enzyme (aminopeptidase) activity	relatively nontoxic; multiplex with other assays, short incubation time (30–60 min)	fluorescence interference with tested compound	Niles et al., 2009 [62]
BrdU (bromoeoxyuridin) and EdU (5-ethynyl-2 deoxyuridine)	DNA synthesis	rapid, highly selective, results in the most reliable and direct index of proliferation, in contrast to ^3^H-thymidine incorporation assay, which requires a scintillation beta-counter, BrdU and EdU can be detected by antibodies, allowing analysis by flow cytometry or by immuno-histochemistry	BrdU is toxic and mutagenic, alters cell cycle, required DNA denaturation	Sidman et al., 1959 [107] Miller and Nowakowski 1988 [108] Salic and Mitchison 2008 [109] Nowakowski et al. 1989 [110] Taupin 2007 [111] P.L Duque and Rakic 2011 [112]

^1^ Alamar Blue: is a resazurin compound that gets reduced to resorufin and dihydroresorufin in viable cells. It can enter live cells so does not require cell lysis, and is stable in culture media. This assay has the added advantage that it can be measured in both fluorimetric and colorimetric plate readers. Pace et al., reported incubation time dependent cytotoxic effect [104]. Calcein-AM: Calcein-acetoxymethylester is a non-fluorescent dye that is used in both cell viability and apoptosis assays and its lipophilic, allowing easy passage through the cell membrane. Once inside the viable cell, intracellular esterases cleave the ester bonds of the acetomethoxy group, resulting in the formation of a fluorescent anionic and hydrophilic calcein dye. Non-viable cells do not contain active esterases. Also need to consider that: Cu^2+^, Co^2+^, Fe^3+^, Mn^2+^, and Ni^2+^ quench the fluorescent signal from calcein at physiological pH, which means care must be taken to select the appropriate cell culture media.

**Table 6:** Luminometric cell viability and cell cytotoxicity assays ^1^.

Luminometric Assay				
	Principal	Advantage	Disadvantage	References
ATP assay	membrane integrity	the fastest and the most sensitive assay to use; no artefacts; no plate handling step	sensitivity depends on reproducibility of pipetting	Maehara et al., 1987 [113], García et al., 2003 [114], Andreotti et al., [115], Markossian et al., 2004 [65]
Real-time viability assay	metabolic activity	real-time measurement; multiplex	incubation time is cell type and seeding density dependent	Duellman et al., 2015 [116], Markossian et al., 2004 [65]

^1^ ATP content: there are numerously available assays that measure ATP levels as an output, when cells begin to undergo cell death process (apoptosis) or lose membrane integrity, ATP stocks become depleted through the activity of ATPases that concurrently prevent any new ATP synthesis. This leads to a rapid depletion of intracellular ATP levels. Luminescent ATP assays function by lysing cells to release ATP stores, while concurrently inhibiting ATPases. Luciferase catalyzes the oxidation of luciferin to oxyluciferin in the presence of magnesium and ATP, resulting in a luminescent signal that directly correlates with the intracellular ATP concentration.

**Table 7:** Cell viability assays for flow cytometry and microscopic imaging ^1^.

Cell Viability Assays for Flow Cytometry and Microscopic Imaging					
		λex	Advantage	Disadvantage	References
Nucleic acid dies	Propidium iodide	488 and 561 nm	can be added directly to the samples	not membrane permeable to live cells, not possible to use on fixed cells, can also bind to RNA not only to DNA, toxigenic and mutagenic	Suzuki et al., 1997 [117]
	7-amino-actinomycin-D (7-AAD)	488 and 561 nm	can be added directly to the samples, can be used in combination with formaldehyde fixation	not membrane permeable to live cells, potential carcinogen	Liu et al., 1991 [118] Latt 1977 [119]
	Hoechst 33342	350 and 454 nm		not membrane permeable to live cells	Liu et al., 2019 [120], Réu et al., 2019 [121]
	Helix NP™ Green, NIR and Blue and SYTOX^®^ based dyes	wide range from 444–640 nm	it can also be used for viability in microscopy on live cells or as a nuclear counterstain on fixed and permeabilized cells and tissue sensitive nucleic acid stain, in combination with resuzarine can be used, works with mammalian and Gram-positive and Gram-negative bacteria, can be incorporated SYTOX^®^ stains into a number of assays for apoptosis, cell viability, and metabolism, easy-to-use.	non cell-permeable to live cells	Avlasevich et al., 2006 [122], Yan et al., 2000 [123], Bryce et al., 2007 [124], Mukhopadhyay et al., 2007 [125]
	DRAQ7™ far-red fluorescent DNA dye	633 nm	can be combined with FITC, PE, and other UV or violet excitable dyes for multicolor analysis, non toxic, can be used for siRNA studies and other dynamic viablity assays	not membrane-permeable to live cells	Kerscher et al., 2019 [126] Paivandy et al., 2019 [127] Vig et al., 2019 [128] Akagi et al., 2013 [129]
	Ethidium bromide	518 nm	can be used on fixed cells, economic	not membrane-permeable, intercalates double-stranded DNA and RNA, mutagen, carcinogen,	Severini and Morgan 1991 [130]
	SYBR^®^ Green	494 nm	highly selective for DNA, can be used in combination with propidium iodide	carcinogen, less mutagenic than ethidium bromide	Zipper et al., 2004 [131], Singer et al., 1999 [132]
	Acridine orange	500 nm	cell permeable, low-cost, sensitive, rapid, intercalate to DNA, and electrostatically interact with RNA, sensitive for pH, acidic organells can be also detected, compatible for ethidium bromide and propidium iodide	nonfixable	Mirrett 1982 [133], Kumar et al., 2012 [134], Darzynkiewicz et al., 2004 [135]
Protein binding dies	eFluor fixable dyes	wide range from 401–645 nm	traditional organic fluorescence dies, multiple application, fully compatible with most convential dies		Lekishvili et al., 2018 [136]
	BD Horizon Brilliant dyes		polymer dyes with brighter fluorescence signal		BD Biosciences products [137]
	Biolegend Zombie dyes	wide range from 360–633 nm			Pardo-Garcia et al., 2015 [138], McMaster et al., 2015 [139], Rodríguez- Rodríguez et al., 2015 [140], Files et al., 2015 [141], Akabane et al., 2016 [142], Iraolagoitia et al., 2016 [143], Mercer et al., 2016 [144], Souza-Fonseca-Guimaraes et al., 2015 [145], Matsui et al., 2015 [146], Jones et al., 2015 [147], Nath et al., 2015 [148], Kanemaru et al., 2015 [149], Tabalot-Ayer et al., 2015 [150], Keppel et al., 2015 [151], Shade et al., 2015 [152], Weiser et al., 2015 [153]
	Calcein AM	496	cell permeant, indicator of lipid vesicle leakage, neutral substrate for MDR efflux transporters, selective for live cells; suitable for proliferating and non-proliferating cells; ideal for suspension and adherent cells, rapid, ideal for high-throughput assays; commonly used for cell tracing and in studies of endocytosis, cell migration, and gap junctions; adaptable to a wide variety of techniques, including: microplate assays, immunocytochemistry, flow cytometry, and in vivo cell tracking		Allen and Cleland 1980 [154], Patel et al., 2009 [155], Glavinas et al., 2004 [156]

^1^ Calcein-AM: Calcein-acetoxymethylester is a non-fluorescent dye that is used in both cell viability and apoptosis assays and is lipophilic, allowing easy passage through the cell membrane. Once inside the viable cell, intracellular esterases cleave the ester bonds of the acetomethoxy group, resulting in the formation of a fluorescent anionic and hydrophilic calcein dye. Non-viable cells do not contain active esterases. Also need to consider that: Cu^2+^, Co^2+^, Fe^3+^, Mn^2+,^ and Ni^2+^ quench the fluorescent signal from calcein at physiological pH, which means care must be taken to select the appropriate cell culture media. Propidium Iodide/7-AAD: These intercalating agents are frequently used to study the cell cycle and they are membrane-impermeable, they are excluded from viable cells. This means that the fluorescence signal emitted by PI or 7-AAD in non-viable cells can be measured either by fluorescence microscopy or FACS analysis. Cell-impermeable DNA-binding dyes such (DRAQ7 from Abcam, Cambridge, UK or SYTOX from Thermo Fisher, Waltham, MA, USA) enter cells through compromised cell membranes and display strong fluorescence upon binding with DNA.

**Table 8:** Conventional assay systems to quantify cell migration.

Migration Assays				
	Principal	Advantage	Disadvantage	References
Transwell migration and invasion assay (Boyden chamber)	chemotaxis, migration, invasion	easy setup, most frequently used method, can be visualized by cytological dyes, stained fluorescent, or lysed and assessed by a plate reader; invasive index can be calculated	endpoint measurement, only adherent cells can be used, only vertical movement can be detected, must be optimized for each cell type	Menyhárt et al., 2016 [64], Kramer et al., 2013 [163], Boyden 1962 [164], Restouin et al., 2009 [165], Marshall 2011 [166]
Scratch (wound healing) assay	two dimensional (2D) cell migration in confluent, monolayer cell cultures	simple, easy setup, low cost, kinetic measurement, can be combined with other techniques (e.g., gene transfections), allows high -throughput screening (HTS)	only adherent cells can be used, only horizontal movement can be detected, require relatively large cell and reagent quantities, is not ideal for chemotaxis studies	Menyhárt et al., 2016 [64], Liang et al., 2007 [167], Győrffy et al., 2015 [168], Gorshkova et al., 2008 [169], Lo et al., 1995 [170], Tamada 2007 [171], Zordan et al., 2011 [172], Poujade et al., 2007 [173], Simpson et al., 2008 [174]
Cell exclusion zone assays	migration	Several kinds of barrier can be used (e.g., glass, silicone, metal, Teflon, microfabricated soft and elastic “stencils” or agarose gels), cells are visualized several manner (e.g., photomicrography or labeled with fluorescence and measured with a microplate reader), migratory capacity and interaction between two different populations can be compared, kinetic measurement, this is currently the only method that allows investigation of the effects of ECM proteins on cell motility, allows high -throughput screening (HTS)	only adherent cells can be used, only horizontal movement can be detected	Menyhárt et al., 2016 [64], Poujade et al., 2007 [173], Gough et al., 2011 [175], Pratt et al., 1984 [176] Varani et al., 1978 [177] Kroening 2010 [178]
Microcarrier bead and spheroid migration assays	migration	adherent cells and spheroids can be used, vertical and horizontal movement, plastic surface and beads can be used, microscope, time-lapse microscope and fluorescent staining can be used to count the cells, endpoint and kinetic measurement can be performed		Menyhárt et al., 2016 [64], Rosen et al., 1990 [179], Konduri et al., 2001 [180]
The capillary chamber migration assay	migration and morphology	adherent and suspension cells can be used, migratory behavior and morphological responses can be visualized in real time by time-lapse microscopy, endpoint and kinetic measurement can be performed, suitable for rare cell types and expensive compounds due to its small cell and volume require, liquid handling and image processing can be fully automated, allows high- throughput imaging system (HTIS)	only horizontal movement can be detected	Menyhárt et al., 2016 [64], Chaubey et al., 2011 [181], Echeverria et al., 2010 [182]
Motility of individual cells	migration	adherent and suspension cells can be used, time-lapse video microscopy, 3D tracking is also possible, analysis of invasive properties of single cells as well as of populations can be performed, kinetic measurement	only horizontal movement can be detected, requires specialized microscopes and image analyzing software	Menyhárt et al., 2016 [64], Miura 2005 [183], Gu et al., 2007 [184], Lin et al., 2005 [185], Niinaka et al., 2001 [186]
Mechanical properties	migration and invasion in 3D structures	3D force microscope and coated magnetic beads can be also used, high-speed video camera, video spot tracker can be applied	only adherent cells can be tested, only horizontal movement can be detected, endpoint assay	Menyhárt el al. 2016 [64], Swaminathan et al., 2011 [187], Cribb et al., 2016 [188]

**Table 9:** Effects of plant-derived natural compounds on cell adhesion and movement. The table summarizes the applied methods and techniques as well.

Active Substance	IUPAC Name (CAS Number)	Source	Cellular Effect	Effect Type	Molecular Mechanisms	Tested Cell Line ^1^, Animal	Method	Ref.
Cistifolin 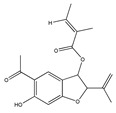	5-acetyl-6-hydroxy-2-(prop-1-en-2-yl)-2,3-dihydrobenzofuran-3-yl (E)-2-methylbut-2-enoate (31685-99-1)	Eupatorium purpureum (rhizome) 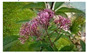	Inhibition of cell adhesion	No data	Modulation of integrin-dependent cell-cell and cell-protein interaction	U937 EA.hy926	Chromatography, Homotypic cell aggregation assay, Cell attachment assay, Carrageenan oedema test	Habtemariam. 1998 [58]
Ursolic acid (UA) 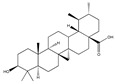	(3β)-3-Hydroxyurs-12-en-28-oic acid (77-52-1)	Berries and wax-like coatings of apples, pears, etc. 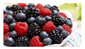	Inhibition of cancer cell adhesion, invasion and migration. Preventing cancer metastasis. Inhibition on proliferation. Inhibition of cell invasion	P	ICAM-1 downregulation	MHCC-97H MHCC-97L HepG2 M619 MDA-MB-231v MCF-7 HT29 SW620 B16-F10 Sprague Dawley rats (males)	MTT cell viability assay, Adhesion assay (fluorescence microscope photographed method), Invasion assay (matrigel coated transwell inserts), Wound healing assay, Flow cytometry, Western blot, Microarray assay, Real-time PCR, *In vivo* tumor growth and metastasis assay, ICAM-1 immunohistochemistry UPLC-MS/MS	Xiang et al., 2015 [229]
Ellagic acid 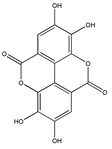	2,3,7,8-tetrahydroxychromeno[5,4,3-cde]chromene-5,10-dione (476-66-4)	*Juglans regia* 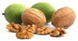	Antiinflammatory activity and osteoblastic activity	P	Inhibition of the effect on TNF-α-induced VCAM-1 and ICAM-1 expression	HAEC KS483	HPLC, Cell-enzyme-linked immunosorbent assay (cell-ELISA), MTT assay	Papoutsi et al., 2007 [18]
Eugenol 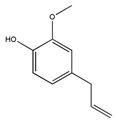	4-allyl-2-methoxyphenol (97-53-0)	*Syzygium aromaticum* 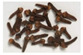	Inhibition of trophozoite adhesion. Anti-*Giardia* activity	No data	No data	*Gardia lamblia* (WB strain [ATCC 30957])	GC, GC/MS, Light microscope, Neubauer cell-counter chamber, Morphological assay, Transmission and scanning electron microscopy, MTT assay	Machado et al., 2011 [230]
Elenolic acid, 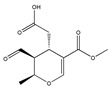	2-[(2S,3S,4S)-3-formyl-5-methoxycarbonyl-2-methyl-3,4-dihydro-2H-pyran-4-yl]acetic acid (34422-12-3)	*Olea europaea* and *Vitis vinifera* 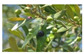	Decreased monocytoid cell adhesion to HUVECs. Anti-inflammatory activity	P	VCAM-1 down-expression	HUEVEC BAEC U937	Enzyme immunoassay (EIA), Cell number and viability assessment (Trypan blue,) Adhesion assay, Northern analysis, Transfection assays (calcium phosphate precipitation method), Electrophoretic mobility shift assay (EMSA)	Carluccio et al., 2003 [21]
Hydroxytyrosol, 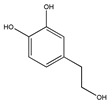	4-(2-hydroxyethyl)benzene-1,2-diol (10597-60-1)							
Oleuropein, 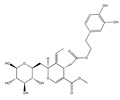	(2S,3E,4S)-3-ethylidene-2-(β-D-glucopyranosyloxy)-3,4-dihydro-5-(methoxycarbonyl)-2H-pyran-4-acetic acid, 2-(3,4-dihydroxyphenyl)ethyl ester (32619-42-4)							
Resveratrol 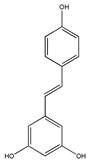	5-[(E)-2-(4-hydroxyphenyl)ethenyl]benzene-1,3-diol (501-36-0)							
Tyrosol, 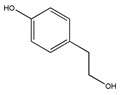	4-(2-hydroxyethyl)phenol (501-94-0)							
Epigallocatechin-gallate (EGCG) 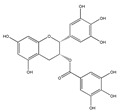		*Camellia sinensis* 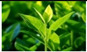	Inhibition of cancer cell adhesion	B	EGCG forms multilayers in the PLL-g-PEG–RGD chains, bocking the RGD motifs	HeLa	Epic BT (RWG label-free biosensor)	Peter et al., 2017 [50]
	(2R,3R)-5,7-dihydroxy-2-(3,4,5-trihydroxyphenyl)chroman-3-yl 3,4,5-trihydroxybenzoate (989-51-5)		Inhibition of cancer cell movement (motility, average motility speed and migration)	No data	No data	HeLa	HoloMonitor M4	Peter et al., 2015 [195]
			Inhibition of cancer cell adhesion to laminin	B	Binding of EGCG to laminin	B16	Trypan blue dye exclusion assay Affinity chromatography	Suzuki and Isemura, 2001 [231]
Curcumin 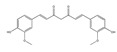	(1E,6E)-1,7-bis(4-hydroxy-3-methoxyphenyl)hepta-1,6-diene-3,5-dione (458-37-7)	*Curcuma longa* 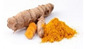	Suppressed monocyte and HUVEC adhesion	P	Down-expression of adhesion molecules. Blocks the NF- κB signaling pathway	HUVEC THP-1	WST-1 colorimetry assay, ELISA, Western Blot	Kawasaki et al., 2015 [13]
			Dose dependent decrease of cell-cell adhesion, especially on tumor-derived spheroids. Decreased cell proliferation in cell lines with mesenchymal characteristics. Decreased the migration speed of highly migratory cells. Decreased tumor growth and aggressiveness.	No data	No data	HACAT NIH-3T3 SCC25 CAL27 HCPA.BALB/c (nude mice)	CyQUANT NF cell proliferation assay kit, Fluorometer, Spheroid assay, Time-lapse analysis (phase microscopy, inverted microscope), Flow cytometry, Xenograft model	Santos de Campos et al., 2017 [232]
Ethyl 3’,4’,5’-trimethoxycinamate (1), 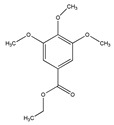	(1) ethyl 3,4,5-trimethoxybenzoate (6178-44-5)	*Piper longum* 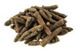	Anti-inflammatory effect. (1) Significantly but reversibly blocked the adhesion of neutrophils to endothelium	P	Down-expression of ICAM-1	Primary endothelial cells from human umblical cord	Trypan blue exclusion test, MTT assay, ELISA, Flow cytometry, NMR, TLC	Kumar et al., 2005 [46]
piperine (2) 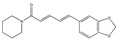	(2) (2E,4E)-5-(benzo[d][1,3]dioxol-5-yl)-1-(piperidin-1-yl)penta-2,4-dien-1-one (94-62-2)					Neutrophil cells from peripheral blood of healthy individuals		
*S*aponin astagaloside IV (AS-IV) 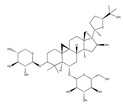	(3β,6α,9β,16β,20R,24S)-16,25-dihydroxy-3-(β-D-xylopyranosyloxy)-20,24-epoxy-9,19-cyclolanostan-6-yl β-D-glucopyranoside (84687-43-4)	*Astagalus membranaceus* 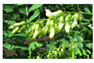	Reduced adhesion of LPS-stimulated HUVECs for PMNs and THP-1 cells	P	Decreased the LPS-induced expression of E-selectin and VCAM-1 on the surface of HUVECs. Decreased the LPS- and TNFα-induced specific mRNA levels for E-selectin and VCAM-1. Abolished LPS- and TNFα-induced nuclear translocation of NF- κB and NF- κB DNA binding activity in endothelial cells	HUVEC THP-1 PMN	ELISA, Northern blot, Electrophoretic mobility shift assay (EMSA), Immunofluorescence staining	Zhang et al., 2003 [22]
Galangin (G) 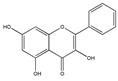	(G) 3,5,7-trihydroxy-2-phenyl-4H-chromen-4-one (548-83-4)	*Alpinia officinarium (G)* 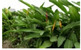	Dose-dependent suppression of U937 cell adhesion to HUVEC monolayer	P	M and Q reduced the IL-1β stimulated expression of VCAM-1, ICAM-1 and E-selectin	HUVEC U937	Trypan blue exclusion test, Ferric-reducing ability assay of plasma method (FRAP), Cytofluor fluorescence multiwell plate reader (dichlorofluorescein), Fluoresent labeling (BCECF-AM), ELISA	Kim et al., 2006 [233]
Kaempferol (K) 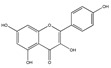	(K) 3,5,7-trihydroxy-2-(4-hydroxyphenyl)-4H-chromen-4-one (520-18-3)	*Gingko biloba,* fruits and vegetables (K) 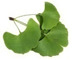						
Myricetin (M) 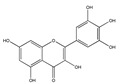	(M) 3,5,7-trihydroxy-2-(3,4,5-trihydroxyphenyl)-4H-chromen-4-one (529-44-2)	Berries, *Camellia sinensis,* etc. *(M)* 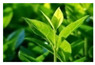						
Quercetin (Q) 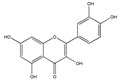	(Q) 2-(3,4-dihydroxyphenyl)-3,5,7-trihydroxy-4H-chromen-4-one (117-39-5)	*Vitis vinifera, Camellia sinensis,fruits* and *vegetables (Q)* 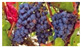						
Baicalein 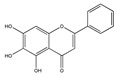	5,6,7-trihydroxy-2-phenyl-4H-chromen-4-one (491-67-8)	*Scutelaria baicalensis* 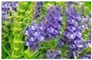	Suppressed MDA-MB-231 cell adhesion to fibronectin-coated substrate	P	Down-regulated the expression of MMP-2/9 involved MAPK signaling pathway	MDA-MB-231	MTT assay, Wound healing assay, Invasion assay, Gelatin zymography, Western blot	Wang et al., 2010 [48]
Fucoidan 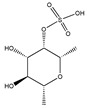	(2S,3S,4S,5S,6R)-4,5-dihydroxy-2,6-dimethyltetrahydro-2H-pyran-3-yl hydrogen sulfate (9072-19-9)	*Ascophyllum nodosum* 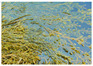	Inhibits cell adhesion to fibronectin	B	Binds to fibronectin with high affinity. Binds to MDA-MB 231 cell membrane and is internalized. Compromises α_5_ subunit distribution on MDA-MB-231 cell membrane	MDA-MB-231	Western blot, Flow cytometer, Scatchard plot analysis, Fluorescence microscopy, Immunofluorescence and confocal laser imaging, Lab-tek chamber	Liu et al., 2005 [234]
Butein 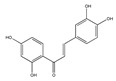	(E)-1-(2,4-dihydroxyphenyl)-3-(3,4-dihydroxyphenyl)prop-2-en-1-one (487-52-5)	*Semecarpus anacardium,* 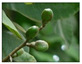 *Dalbergia odorifera,* 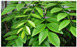 *Caragana jubata,* 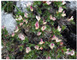 *Rhus verniciflua* 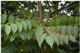	Significantly decreased TNF-α -induced U937 cell adhesion to lung epithelial cells in a dose dependent manner. Anti-inflammatory	P	Inhibition of TNF-α-induced ICAM-1 and VCAM-1 expression by inhibiting the NF- κB/ MAPK/Akt signaling pathway. Inhibited expression of ICAM-1 and VCAM-1	A549 U937	Western blot, Fluorescent labeling (BCECF-AM), RT-PCR, Luciferase reporter gene assay, Fluorescence microscopy, Fluocytometer (H_2_DCFDA)	Jang et al., 2012 [49]
*Tripterygium wilfordii extract* (TWH-f), and tetrandrine 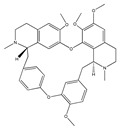	(1β)-6,6’,7,12-tetramethoxy-2,2’-dimethylberbaman (518-34-3)	*Tripterygium wilfordii* 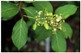	Affects the secretion and expression of adhesion molecules on cells	P	TWH-f at high concentration has significant inhibitory effect on expression and secretion of E-selectin, ICAM-1, VCAM-1. Tetrandrine did not demonstrate the same effects.	Neutrophils HUVEC HFB	Flow cytometry, ELISA	Chang et al., 1999 [7]
„Danshen” (DS)		*Salvia miltiorrhiza (DS)* 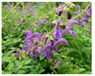	SZ and HSW significantly inhibited apoptosis in HUVEC undergoing serum deprivation and TNF-α stimulation	P	Down-regulation of caspase-3-gene expression. DS and SQ significantly attenuated TNF-α-induced expression of VCAM-1 and ICAM-1	HUVEC	Annexin-V staining, Western blot, Flow cytometry, [^3^H]-thymidine incorporation assay	Ling et al., 2007 [2]
„Sanchi” (SQ)		*Panax notoginseng* (SQ) 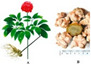						
„Shanzai” (SZ)		*Crataegus* (SZ) 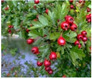						
„Heshouwu”(HSW)		*Polygonum multiflorum (HSW)* 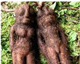						

^1^ U937: human monocyte leukemia cells, EA.hy926: human somatic cell hybrid endothelial cells, MHCC-97H: human hepatocellular carcinoma, MHCC-97L: human hepatocellular carcinoma, HepG2: human hepatocellular carcinoma, M619: human uveal melanoma, MDA-MB-231: human breast adenocarcinoma, MCF-7: human breast adenocarcinoma, HT29: human colorectal adenocarcinoma, SW620: human colorectal adenocarcinoma, B16-F10: mouse melanoma cells, HAEC: human aorta endothelial cells, KS483: murine preosteoblastic cells, HUVEC: human umbilical vein endothelial cells, BAEC: bovine aortic endothelial cells, U937: human monocyte leukemia cells, HeLa: human cervix epithelial adenocarcinoma, THP-1: human monocytic cells, PMN: polymorph-nuclear leukocytes, A549: human lung epithelial cells, HACAT: human spontaneously transformed aneuploid immortal keratinocyte cell line, NIH-3T3: mouse fibroblast, SCC25: human oral squamous cell carcinoma, CAL27: oral adenosquamous carcinoma cell line, HCPA.BALB/c (nude mice), HFB: human synovial fibroblast, B16: mouse melanoma cell line.

**Table 10:** Effects of animal-derived natural compounds on cell adhesion and movement. The table summarizes the applied methods and techniques as well.

Active Substance	Source	Cellular Effect	Effect Type	Molecular Mechanisms	Tested Cell Line ^1^, Animal	Method	Ref.
Rhodostomin 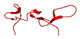 Disintegrin 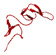	*Calloselasma rhodostoma* (venom) 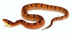	Cell detachment. Actin cytoskeleton perturbed	B, P	Prevention of integrin α_v_β_3_ interaction. Decreased pp125^FAK^ phosphorylation	HUVEC	Cell detachment assay (Trypan blue), MTT assay, Flow cytometry, DNA fragmentation assay, Precipitation of cell extracts and immunoprecipitation, Western blot, Immunofluorescence microscopy	Wu et al., 2002 [55]
Echistatin 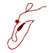	*Echis carinatus* (venom) 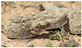	Decreased cell adhesion	B	Prevention of α_IIb_β_3_, α_v_β_3_ and α_5_β_1_ integrin interactions	HeLa	Epic BT (RWG label-free biosensor)	Szekacs et al., 2018 [51]

^1^ HUVEC: human umbilical vein endothelial cells, HeLa: human cervix epithelial adenocarcinoma.

## Data Availability

All relevant data are available in the manuscript.
